# Efficient *in planta* production of amidated antimicrobial peptides that are active against drug-resistant ESKAPE pathogens

**DOI:** 10.1038/s41467-023-37003-z

**Published:** 2023-03-16

**Authors:** Shahid Chaudhary, Zahir Ali, Muhammad Tehseen, Evan F. Haney, Aarón Pantoja-Angles, Salwa Alshehri, Tiannyu Wang, Gerard J. Clancy, Maya Ayach, Charlotte Hauser, Pei-Ying Hong, Samir M. Hamdan, Robert E. W. Hancock, Magdy Mahfouz

**Affiliations:** 1 Laboratory for Genome Engineering and Synthetic Biology, Division of Biological Sciences, 4700 King Abdullah University of Science and Technology (KAUST), Thuwal, 23955-6900 Saudi Arabia; 2 Laboratory of DNA Replication and Recombination, Division of Biological Sciences and Engineering, King Abdullah University of Science and Technology (KAUST), Thuwal, 23955-6900 Saudi Arabia; 3 Centre for Microbial Diseases and Immunity Research, Department of Microbiology and Immunology, University of British Columbia, Vancouver, BC Canada; 4 Laboratory for Nanomedicine, Division of Biological and Environmental Science and Engineering, King Abdullah University of Science and Technology (KAUST), Thuwal, 23955-6900 Saudi Arabia; 5 Biochemistry Department, Faculty of Science, University of Jeddah, Jeddah, 21577 Saudi Arabia; 6 Water Desalination and Reuse Center, Division of Biological Sciences and Engineering, King Abdullah University of Science and Technology (KAUST), Thuwal, 23955-6900 Saudi Arabia; 7 Analytical Chemistry Core Laboratory, King Abdullah University of Science and Technology (KAUST), Thuwal, 23955-6900 Kingdom of Saudi Arabia; 8 Imaging & Characterization Core Laboratory, King Abdullah University of Science and Technology (KAUST), Thuwal, 23955-6900 Kingdom of Saudi Arabia

**Keywords:** Molecular engineering in plants, Recombinant peptide therapy, Genetic engineering, Molecular engineering in plants

## Abstract

Antimicrobial peptides (AMPs) are promising next-generation antibiotics that can be used to combat drug-resistant pathogens. However, the high cost involved in AMP synthesis and their short plasma half-life render their clinical translation a challenge. To address these shortcomings, we report efficient production of bioactive amidated AMPs by transient expression of glycine-extended AMPs in *Nicotiana benthamiana* line expressing the mammalian enzyme peptidylglycine α-amidating mono-oxygenase (PAM). Cationic AMPs accumulate to substantial levels in PAM transgenic plants compare to nontransgenic *N. benthamiana*. Moreover, AMPs purified from plants exhibit robust killing activity against six highly virulent and antibiotic resistant ESKAPE pathogens, prevent their biofilm formation, analogous to their synthetic counterparts and synergize with antibiotics. We also perform a base case techno-economic analysis of our platform, demonstrating the potential economic advantages and scalability for industrial use. Taken together, our experimental data and techno-economic analysis demonstrate the potential use of plant chassis for large-scale production of clinical-grade AMPs.

## Abstract (web summary)

Antimicrobial peptides (AMPs) are next-generation antibiotics that can be used to combat drugresistant pathogens. Here, the authors report efficient production of bioactive amidated AMPs by transient expression in *Nicotiana benthamiana* line expressing the mammalian enzyme peptidylglycine α-amidating mono-oxygenase.

## Introduction

Against the backdrop of emerging antimicrobial resistance in multiple human pathogens, antimicrobial peptides (AMPs) and host defense peptides (HDPs) hold considerable promise for clinical applications. AMPs evolved as part of the immune systems in many species and kill bacterial cells (including drug-resistant strains)^1^ by interacting with their membranes followed by multimodal mechanisms that can include membrane perturbation, inhibition of cell wall synthesis and inhibition of internal targets, including synthesis of macromolecules^2,3^, acting more rapidly than classical antibiotics^4^, and limiting the evolution of drug resistance^5,6^. AMPs can also exhibit potent activity against bacterial biofilms, independent of AMP activity^7^, and diverse immunomodulatory effects^8^. The pervasive collateral sensitivity of AMPs towards drug-resistant bacterial strains^9^, and their marked functional synergism with current antibiotics^10^ underscores their potential use as effective therapeutic drugs. However, despite having myriad applications in clinical medicine, the development of clinically translated AMPs has only recently begun to accelerate.

Nevertheless, significant progress is being achieved with respect to peptide technology and medicine in the form of three major waves over the last fifty years, which has taken a further leap by innovative computational technologies and artificial intelligence^11^. Several peptide drugs have been approved by the US Federal Drug Administration (FDA) such as the human immunodeficiency virus (HIV) fusion inhibitor called fuzeon, which forms a part of highly active antiretroviral therapy regimen^12^. The approval of peptide use has opened avenues for multi-faceted, peptide-based systems in the treatment of different diseases and their commercial use^13^. However, AMPs are particularly challenging and costly to manufacture synthetically, slowing down their clinical translation. Conventional AMP manufacturing relies on solid-phase peptide synthesis (SPPS) with a cost between $100 and $600 per gram^2^, although certain efficiencies can be gained by optimizing large-scale synthesis that help bring down costs. Moreover, solid-phase peptide synthesis suffers from the prohibitive limitation of peptide length, which should be no more than 50 amino acids^14^, the presence of hydrophobic peptides that tend to aggregate in the solvents used for synthesis^15^, and the need to use hazardous chemicals and solvents throughout the peptide synthesis and purification procedures^16^.

Synthetic biology offers the promise of sustainable, scalable, and cost-effective production of AMPs, based on genetically engineered organisms^17^. The optimization of growth medium for *E. coli* with the Notomista-Arciello broth^18^ resulted in unprecedented gains in cell biomass and AMP production capacity. Indeed, increases in production scale of the recombinant AMPs ONC-r(P)GKY20, ONC-r(P)ApoBL, and ONC-r(P)ApoBS increased yield from 100 mg to 1000 mg per batch, while dropping the cost from €253 to €42 per mg. Another study used genetically engineered yeast (*Pichia pastoris*) for the heterologous production of recombinant AMPs in bioreactors, yielding 1 g of AMPs at a minimum cost of 1 US$^19^. AMPs produced in both studies exhibited the same biological activity as their synthetic counterparts, bypassing the cost issues and presenting robust biomanufacturing platforms for AMP production.

Although AMPs can be produced in bacterial or yeast cells and purified to homogeneity, the use of plants as a production host for complex biologics is deemed safer, demands less infrastructure, and has the potential for rapid scaling-up of production^20^. In fact, producing proteins in plants is estimated to cost 10- to 50-times less than *E. coli* fermentation^21^. However, *in planta* production of peptides has proven difficult, presumably due to proteolysis by plant proteases. To circumvent this incompatibility, various strategies have been deployed, such as the downregulation of genes encoding interfering plant proteases^22^ or restricting AMP production to a specific organelle^23^. Despite these strategies, the typical yields from plant-produced peptides have generally been low^24,25^.

The clinical application of AMPs is also hindered by their contextual activity^26^, as they are readily cleaved by proteases^27^, are poorly tolerant to acidic pH environments^28^, and exhibit poor stability in serum^8,29^, thereby creating an unfavorable pharmacokinetic profile. These limitations often result in peptides with a low therapeutic index, which has restricted the clinical regimen involving AMPs to topical applications^30^. To circumvent these shortcomings, the pharmacokinetic/pharmacodynamic properties of AMPs should be improved to achieve the same clinical outcomes as conventional antibiotics. For example, the incorporation of D-amino acids into the peptide backbone has been shown to impair the proteolytic degradation of AMPs by proteases^28^. The de novo engineering of cationic AMPs as amphipathic helices has also shown potential for clinical translation^31,32^, in addition to conformational constraints via cyclization for improved bioavailability in the gut^33^, orthogonal screening of iterative peptide libraries^34^, and C-terminal amidation^35^ to tailor AMPs (especially shorter peptides) with superior antimicrobial potency and efficacy.

Amidation is a highly conserved post-translational modification that is often required for many glycine-extended peptide hormones to exert their full biological activity in vivo^36^. Amidation of AMPs has been suggested to enhance their electrostatic binding to the negatively charged bacterial membrane^37^, improve their potency^38^ and prevent their enzymatic degradation^39^ and is critical in shorter peptides (<~15-amino acids). In mammals, the bifunctional peptidylglycine α-amidating mono-oxygenase (PAM) enzyme catalyzes the two-step conversion of peptidylglycine substrates into α-amidated products, in the presence of the co-factors copper, ascorbic acid and molecular oxygen^40^. The first step in this conversion is an oxidation mediated by the enzymatic PHM (peptidylglycine α-hydroxylating monooxygenase) domain of PAM that hydroxylates the C-terminal glycine extension; the second step is mediated by the PAL (peptidyl-α-hydroxyglycine α-amidating lyase) domain that promotes the cleavage of the resulting α-hydroxylated peptide intermediate, yielding the α-amidated product^40^.

In this work, we describe a unique approach for the control production of amidated AMPs by a targeted combination of transgenic and transient expression modules in *N. benthamiana*. We use PAM enzymes from rats (*Rattus norvegicus*) to introduce the mammalian C-terminal amidation pathway into *N. benthamiana* plants. For transient production of AMPs, we select the amino acid sequences of three synthetic HDPs that have demonstrated potent antimicrobial, antibiofilm and immunomodulatory activity profiles against drug-resistant pathogens in vitro and in vivo^7,41,42^. Specifically, the peptides are 1018-G (VRLIVAVRIWRRG), 1002-G (VQRWLIVWRIRKG), and 3002-G (ILVRWIRWRIQWG) designated as AMP1, AMP2 and AMP3 respectively, which differ from the parent sequences by having an additional Gly at the C-terminus. We demonstrate the successful production of both the nonamidated Gly precursors as well as the final PAM processed amidated AMP counterparts. Moreover, we reach a titer for these amidated peptides of 1.4 mg per 20 g of transgenic plant tissue. Importantly, these purified peptides are biologically active against ESKAPE pathogens, with low collateral toxicity towards mammalian cells. Overall, our results highlight the exceptional flexibility of plant-based production platforms for potential large-scale production of amidated AMPs.

## Results

### Design of constructs for *in planta* production of AMPs

**Fig. 1:**
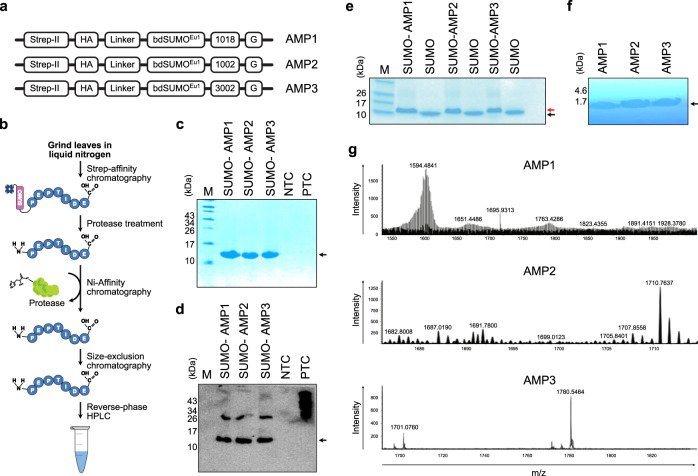
Establishment of a SynBio chassis for *in planta* expression of AMPs. **a** Schematic diagram of the AMP expression cassette for *in planta* expression, using the backbone of the pEAQ-HT vector. Strep-II, high-affinity strep-tag II; HA, human influenza hemagglutinin epitope; linker, flexible GGSGGS linker; bdSUMOEu1, small ubiquitin-related modifier (bdSUMO) from Brachypodium distachyon containing mutations at SUMO-interacting positions; AMP1, AMP2 and AMP3 with a terminal glycine residue. **b**, Flowchart created using Affinity designer (https://affinity.serif.com/en-us/) summarized the plant-based production and purification of biologically active AMPs. The individual plasmids are transformed into Agrobacterium tumefaciens and infiltrated into Nicotiana benthamiana; leaves are harvested at 6 days post infiltration (dpi); total proteins are isolated and applied to Strep-Tactin Superflow resin. Following protease cleavage, His-tagged SENPEuH is removed using Ni-affinity chromatography and isolated AMPs are further purified by size-exclusion chromatography (SEC). Next, the pooled SEC fractions are applied to a reverse-phase high-performance liquid chromatograph (RP-HPLC) for final purification of AMPs. **c** Analysis of the isolated proteins by SDS-PAGE. Strep-II affinity-captured SUMO-fused AMPs were separated by SDS-PAGE and gels were stained with Coomassie Brilliant blue. **d** Immunoblot confirmation of purified SUMO-fused AMPs. The separated proteins were transferred onto a polyvinylidene difluoride membrane and probed with a monoclonal anti-HA antibody for detection of bdSUMO^Eu1^-AMPs (∼15.5 kDa). Total proteins extracted from non-infiltrated leaves serving as negative control, NTC; HA-tagged protein used as a positive control, PTC. Two independent blots were performed with similar results. Black arrowheads indicate the expected size of protein. **e** Gel shift assay for AMP release. Proteins were separated on a 18% Tricine-SDS gel to detect the release of AMPs peptide (∼1.5–1.7 kDa) from the bdSUMOEu1 domain (∼14 kDa). Red and black arrowheads indicate the uncleaved and cleaved proteins, respectively. **f** RP-HPLC purification of AMPs. Pooled fractions from SEC were separated on C8 column using acetonitrile gradient. Purified AMPs were separated on a 18% Tricine-SDS gel. Two independent Tricine-SDS-PAGE gels were performed with similar results. **g** Mass analysis of plant-purified AMPs using ESI-MS. The y-axis shows the signal intensity, and the x-axis displays the m/z value of each peptide. Black arrowheads indicate the expected size of peptides. Source data are provided as a Source Data file.

To date, few successful examples have reported using plants as a platform for large-scale production of AMPs^43–45^, perhaps reflecting an inherent incompatibility between the plant host and the heterologous peptides to be produced^46^. Here we leveraged synthetic biology to unlock the potential of *N. benthamiana* as a cost-effective chassis for large-scale production of hydrophobic-rich, cationic-charged AMPs. To ensure high-efficiency purification, we added the eight amino-acid *Strep*-tag II sequence to the AMPs for high-affinity binding to the engineered streptavidin *Strep*-Tactin^47^ (Fig. 1a). An elastin-like polypeptide (ELP) tag was previously employed for purification of AMPs in plants^46^. However, even though the ELP tag is inert, a tag or residual sequences in the final peptide backbone may alter the structural conformation of the final AMPs and reduce their overall efficacy. Therefore, we chose to use inert protein tags juxtaposed by cleavage sequences for the tag release in our engineered peptide constructs. We also opted for a cytosolic accumulation to avoid adding a targeting signal before the desired AMPs. Lastly, we added the flexible linker (GGGSGGGS) to preserve the functionality of the fused protein, by allowing independent movement of the N and C portions (Fig. 1a).

The prototypic AMPs selected for this study harbor leucine and arginine residues at their N termini, which would normally make them more susceptible to protease degradation via the N-end rule pathway^48,49^. Moreover, AMPs can take on the characteristics of a signal peptide due to their high hydrophobicity and strong positive charge^50^, and are prone to degradation by the proteases of the secretory pathway between the endoplasmic reticulum (ER) and the Golgi apparatus^51^. Here, we added an engineered version of the plant SUMO (Small Ubiquitin-like Modifier) domain termed SUMO^Eu1^ to the N terminus of the target amino acid sequences of the HDP to increase peptide stability and solubility in the plant cytosol while also ensuring exact cleavage site production without extra residues. While eukaryotes possess endogenous SUMO proteases that can cleave SUMO-tagged proteins in vivo^52^, the SUMO^Eu1^ domain contains three amino acid changes that render it resistant to degradation except by its cognate SUMO-specific protease, SENP^EuH^^53^. Importantly, this cleavage reaction leaves no residual amino acids^54–56^, thus allowing release of AMPs in their native form, concomitantly averting the elution of non-specific background binders^53^. Moreover, the protease was previously reported to be efficient in cleaving domains of proteins immobilized on cellulose beads in vitro or within the confined environment of cells in vivo^57^, thereby demonstrating the robustness and precise activity of protease. We also added a C-terminal glycine residue to all AMPs as a substrate for eventual PAM-mediated amidation (Fig. 1a).

We introduced all elements into the plant vector pEAQ-HT carrying parts of genomic RNA2 from cowpea mosaic virus (Fig. 1a), which facilitates hypertranslation of heterologous constructs in plants, and allows the production of AMPs at high titer in the infected leaves^58^. The use of whole-viruses to produce AMPs was previously described in plants^59^ but suffers from several drawbacks in terms of transgene size that can be cloned, and concerns associated with biocontainment^60^. The vector used here can be readily delivered into leaves using *Agrobacterium*, and it does not pose a risk of biocontamination to the environment since it is harboring a deconstructed virus backbone. Notably, pEAQ-HT also harbors a P19 post-transcriptional gene silencing suppressor gene, further enhancing gene expression levels.

### Transient expression and purification of AMPs

The purification protocol summarized in Fig. 1b was utilized. We infiltrated Agrobacteria harboring the *AMP-*expressing constructs into the leaves of *N benthamiana* at an OD_600_ of 0.5. Six days later, we probed total extracts from the infiltrated leaves for protein accumulation by SDS-PAGE (Fig. 1c) and immunoblotting (Fig. 1d). During protein purification, we added polyvinylpolypyrrolidone to sequester phenolic contaminants and prevent unwanted proteolysis with a cocktail of protease inhibitors. Solubilized SUMO^Eu1^-AMPs was purified by *Strep*-tag II affinity chromatography and eluted using 2.5 mM d-desthiobiotin. All three recombinant SUMO-fused peptides were successfully detected in the leaf extracts (Fig. 1c, d). The introduced SUMO^Eu1^ domain facilitated the release of native AMPs from the rest of the produced protein via proteolytic cleavage with SENP^EuH^, as indicated by the mass shift after electrophoresis (Fig. 1e). We immersed the gel directly in an aqueous Coomassie brilliant blue G-250 solution containing 40% methanol and 4% formaldehyde to help retain smaller peptides. To ascertain that the bands were not an artefact of bromophenol blue in the sample loading buffer, we performed protein separation with or without bromophenol blue in the loading dye and obtained the same results (Supplementary Fig. 1a). Histidine-tagged protease was removed from the solution by Ni-affinity chromatography.

The use of low molecular-weight filters to separate the released peptide from uncleaved fusion protein was avoided, since peptides tend to adsorb to filter matrices by ionic and hydrophobic interactions. Instead, size exclusion chromatography (SEC) using organic modifiers was utilized, for two reasons: i) SEC facilitates desalting in contrast to affinity-based chromatography that includes salts that could result in nonspecific matrix interactions with the hydrophobic, cationic peptides, and ii) SEC columns allow buffer exchange into the highly volatile mobile phase buffer for subsequent reverse-phase high-performance liquid chromatography (RP-HPLC) analysis. All three AMP peptides eluted at the earliest stage between 1- and 2-mL at a flow rate of 0.01 mL/min (Supplementary Fig. 1b); all eluted fractions covering the peaks were collected for purification via RP-HPLC, which served as an additional desalting step^61^. We carried out RP-HPLC using acetonitrile as the organic modifier and HCl as ion-pairing agent rather than traditional trifluoro-acetic acid that has inherent toxicity and would need to be exchanged for a biocompatible ion^62^. The three experimental peptides eluted from the column at around 80% acetonitrile between 9–12 min (Supplementary Fig. 1c). Analysis of the fractions on 18% Tricine-SDS-PAG gels revealed single peptide bands for each peptide after staining with Coomassie brilliant blue G-250 solution containing 40% methanol and 4% formaldehyde (Fig. 1f), highlighting the high purity of the peptides. We confirmed peptide masses by ESI-MS based on their expected m/z ratios (1018-G: 1593.01 *m/z*); (1002-G: 1709.0361 *m/z*); (3002-G: 1781.0361 *m/z*) (Fig. 1g). Such peaks were not present in the extract obtained from wild type *N. benthamiana* (Supplementary Fig. 6b).

### PAM enzymes amidated glycine-extended AMPs in plant

**Fig. 2:**
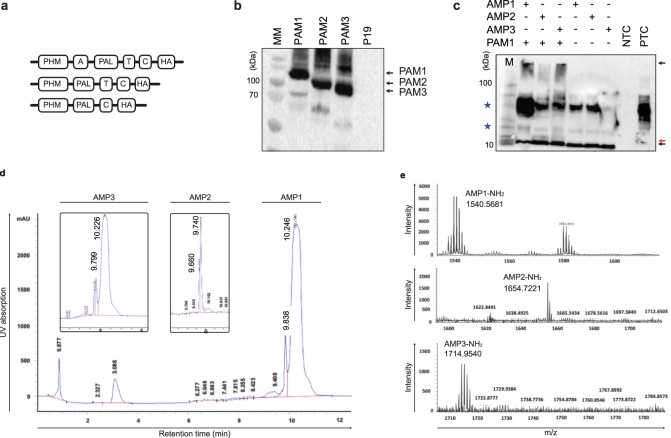
Plant-based platform for production of amidated AMPs. **a** Schematic diagram of chimeric cassettes with different variants of bifunctional rat PAMs and the different domains: PHM, peptidylglycine α-hydroxylating monooxygenase domain; PAL, peptidyl-α-hydroxyglycine α-amidating lyase domain; A, region encoded by exon 16 separating the PHM and PHL domains; T, transmembrane domain; C, cytoplasmic domain. The HA epitope was added for immunodetection of PAMs. **b** *In planta* transient expression of PAM enzymes. Each plasmid was individually co-infiltrated in *N. benthamiana* leaves along with a plasmid encoding P19. Leaves were harvested 3 dpi, total proteins were extracted and analyzed by immunoblotting using a monoclonal anti-HA antibody. The predicted molecular masses were 120 kDa (PAM1), 105 kDa (PAM2) and 95 kDa (PAM3). The three black arrowheads indicate the bands corresponding to PAM1, PAM2, and PAM3 enzymes. Two independent blots were performed with similar results. **c** *In planta* transient co-expression of AMPs and PAM enzymes. Constructs encoding AMPs and PAMs were transiently co-expressed in *N. benthamiana* (1:1 ratio) and the extracts were immunoblotted using anti-HA antibody. Black arrowheads indicate the expected size of proteins and peptides, red arrowheads indicate to possible *in planta* modified AMPs, while the asterisk indicates the non-specific bands. Two independent blots were performed with similar results. **d** *In planta* amidation of AMPs in transgenic plants expressing *PAM1*. Transgenic *N. benthamiana* lines (T_4_ generation) overexpressing a *PAM1* variant were infiltrated with constructs encoding glycine-extended AMPs. AMPs were isolated with our established test method and subjected to separation in RP-HPLC using C8 with an acetonitrile gradient from 20 to 80% in 0.01 M HCl and monitored at the 215 nm wavelength. Purified peptides were eluted as a double peak, with the major peak belonging to amidated peptide with retention times of 10.2 min (AMP1), 9.7 min (AMP2), 10.2 min (AMP3) and the minor peak belonging to the non-amidated form with retention times 9.5 min (AMP1), 9.6 min (AMP2) and 9.7 min (AMP3). **e** Confirmation of AMP amidation via ESI-MS. Mass analysis of purified AMPs isolated from the *PAM* transgenic plants showing major peak belonging to amidated AMPs along the minor non-amidated peak. Source data are provided as a Source Data file.

The *PAM* cDNAs from the rat genome, were then subcloned and expressed individually in *N. benthamiana*. The encoded PAM enzymes had both PHL and PAM domains, and *PAM* transcripts often undergo alternative splicing resulting in either integral membrane-bound (PAM1/2) or soluble (PAM3) forms of the enzyme^63^. The coding sequence of each *PAM* isoform was cloned into the binary vector pK2GW7 and transiently expressed individually in *N. benthamiana* leaves (Fig. 2a). Following confirmation of expression (Fig. 2b), we assessed the ability of each PAM isoform to amidate glycine-extended AMPs *in planta* via co-expression by immunoblotting from total protein extracts using anti-HA antibodies. Specific signals of the expected molecular weights of 15 kDa (for AMPs) and 110 kDa (for PAM1) were observed in extracts from co-infiltrated leaves (Fig. 2c). We also noticed a pattern of retarded bands (indicated by red arrowheads) migrating closely with the AMPs signal (indicated by black arrowheads) in extracts from PAM1 and AMPs co-infiltrated leaves, no such retarded bands pattern were observed in extracts from leaves infiltrated with the *AMP* constructs alone (Fig. 2c). The appearance of extra bands suggests the post-translational modification of AMPs mediated by transiently expressed PAM1 enzyme.

Therefore stable *N. benthamiana* transgenic lines expressing *PAM1* as a source of *in planta* amidation were generated. Importantly, the resulting transgenic *PAM1* lines exhibited no obvious morphological or developmental changes relative to nontransgenic *N. benthamiana*, aside from a considerable decrease in seed yield. We next tested whether these transgenic lines were able to amidate glycine-extended AMPs produced from a transiently infiltrated construct. Following *Agrobacterium*-mediated infiltration of the *AMP* constructs, the three AMPs were cleaved by SUMO protease and purified as above. RP-HPLC analysis detected one major peak and one minor peak at 215 nm for each AMP after separation on a C8 column (Fig. 2d). The peaks were consistent with previous work on the in vitro amidation of a precursor peptide produced in *E. coli*^64^. We estimated the percentage of amidation, as judged by RP-HPLC, to be more than 80% of total AMP abundance. We confirmed the amidation of the peptides by electrospray ionization time-of-flight mass spectrometry (ESI-TOF-MS) analysis, as evidenced by the expected *m/z* ratios (Fig. 2e) (AMP1-1536.93, AMP2- 1653.05, AMP3-1725.12).

Using our established protocol, we also successfully purified other AMPs as SUMO fusions: FK13, YI12, Guavanin 2, WLBU2, CONGA, DBS1, Mastoparan 4,1, and even the green fluorescent protein (GFP) (Supplementary Fig. 1d, e). Interestingly, the amount of peptide produced in the *PAM1* transgenic plants was quite substantial (1.4 mg per 20 g of leaf biomass) relative to that obtained from nontransgenic *N. benthamiana* plants (0.39 mg per 20 g of leaf biomass).It could be possible that C-terminal amidation shields peptides from proteases in transgenic plants, as previously reported for peptides in human serum^65^. The overall yields of individual downstream processing step are also summarized (Supplementary Fig. 1f). Taken together, these results demonstrate that this approach is suitable for plant-based production of AMPs with a defined terminal C-amide residue.

### AMPs exhibited low toxicity towards mammalian cell

**Fig. 3:**
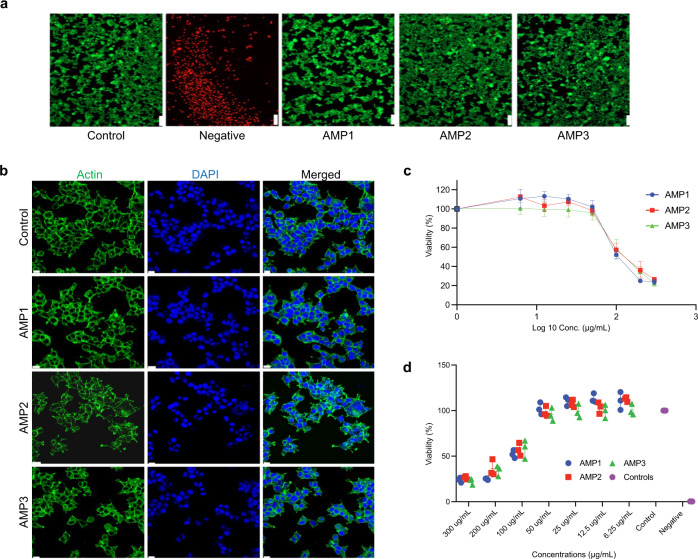
Plant-purified peptides display low toxicity in mammalian cells. **a** Calcein AM (green, live cells) and ethidium homodimer (red, dead cells) staining of HEK293 cells 48 h after treatment with each peptide (50 μg/mL). **b** F-actin (green) and nuclei (blue) were stained with phalloidin-rhodamine/DAPI for the immunofluorescence staining after 72 h of treatment under laser scanning confocal microscope; scale bar: 20 μm. Two independent microscopy experiments have been performed with similar results. **c** Representative dose–response curves and **d** bar graph illustration of metabolic index of both peptide treated (300, 200, 100, 50, 25, 12.5, 6.25 μg/mL) and untreated cells as measured by estimating the cellular ATP levels using luciferase-based CellTiter-Glo® reagent. Cell viability was quantified as the ratio of ATP levels-dependent luminescence in treated / untreated cells expressed as percentage. Untreated: cells in a tissue culture plate treated with PBS; Negative control: cells treated with 1% [v/v] Triton-X 100 (± SD, *n* = 3 independent biological replicates). Source data are provided as a Source Data file.

Recombinant proteins produced in *E. coli*, are generally contaminated with endotoxin, which greatly limits their use as bacterially produced therapeutics^66^. Minor traces of endotoxin impair cellular proliferation^67^ and can induce programmed cell death^68^. While plants contain very low levels of endotoxin^69^, *Agrobacterium* in our system could result in endotoxin contamination, since the bacteria can survive for weeks in the plant intercellular space. AMPs themselves have an inherent risk of collateral toxicity due to their ability to disrupt mammalian cellular membranes^8^, which often needs to be carefully verified when preparing AMP-based therapeutics before clinical studies. We, therefore, tested the biocompatibility of each plant-produced purified AMP on in vitro cultured human embryonic kidney 293 (HEK293) cells. At a concentration of 50 µg/mL, each peptide exhibited no adverse effects on HEK293 cells, as shown by a live/dead cytotoxicity assay (Fig. 3a). Adenosine triphosphate quantification assay further demonstrated no differences in ATP production between all groups, indicating that cell proliferation of treated cells is comparable to the control (Supplementary Fig. 2). Further immunostaining of F-actin filament using fluorescently labeled phalloidin revealed no changes in the morphological characteristics of treated cells thereby asserting the low cytotoxic effect of each peptide on mammalian cells (Fig. 3b). However, mild cytotoxicity was observed at a higher concentration of 100 µg/mL tested for each purified peptide, as shown by the sensitive ATP quantifying assay (Fig. 3d). The estimated IC_50_ value of each peptide were 126.4, 147.9 and 140 µg/mL for AMP1, AMP2 and AMP3 respectively (Fig. 3c). Based on our data, our plant-produced peptides appear to have extremely low (50 µg/mL) to mild cytotoxicity at higher concentrations (100 µg/mL).

### AMPs demonstrated robust killing of ESKAPE pathogens

**Fig. 4:**
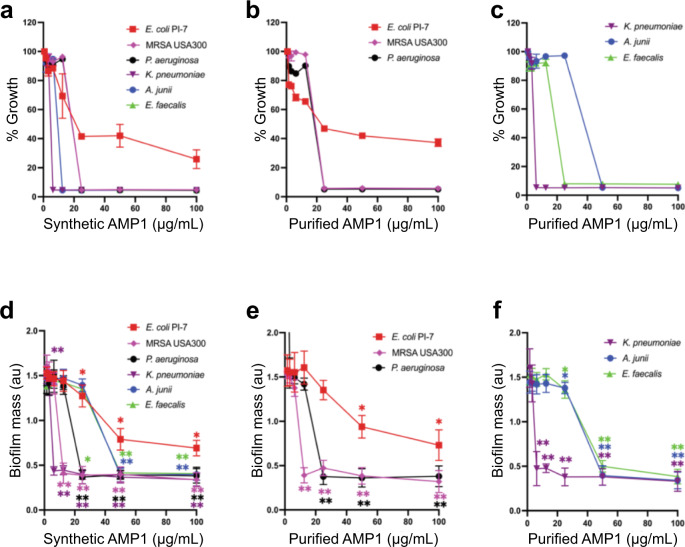
Experimental validation of antimicrobial activity of plant-purified AMP1 against ESKAPE pathogens and their prevention of biofilm formation. **a**–**c**, Purified peptides exhibit a similar efficacy as synthetic peptides. For each assay, 10^6^ colony-forming units (CFU)/mL of each ESKAPE [*E. coli* PI-7, methicillin-resistant *S. aureus* (MRSA) USA300*, K. pneumoniae, A. junii, P. aeruginosa*, and *E. faecalis*] pathogen were treated with 100, 50, 25, 12.5, 6.25, 3.215, 1.56 µg/mL of peptides in cation-adjusted Mueller-Hinton broth for 24 h. Up to >50% reduction of carbapenem-resistant *E. coli PI-7* in OD600, at a concentration of pp: 50 μg/mL, sp: 50 μg/mL, >90% of inhibition of MRSA USA300 (pp: 25 μg/mL, sp: 25 μg/mL), *P. aeruginosa* (pp: 25 μg/mL, sp: 25 μg/mL), *K. pneumoniae* (pp: 6.25 μg/mL, sp: 6.25 μg/mL), *A. junii* (pp: 50 μg/mL, pp: 12.5 μg/mL), *E. faecalis* (pp: 25 μg/mL, sp: 25 μg/mL); pp: purified peptide; sp: synthetic peptide. Data are mean ± SD of three independent experiments performed in duplicates. **d**–**f**, Bactericidal activity of synthetic and purified peptide for the prevention of biofilm formation after 24 h of incubation in biofilm medium containing various concentrations of peptides. Results are expressed as biofilm mass, measured using crystal violet staining, in arbitrary units (au). Data are mean ± SD of three independent experiments performed in duplicates. The purified AMP1 abolish >90% of MRSA USA300 biofilms at 12.5 μg/mL, *P. aeruginosa* at 25 μg/mL, *A. junii* at 50 μg/mL, *K. pneumoniae* at 6.25 μg/mL, *E. faecalis* at 50 μg/mL (*P* = 0.0022). *, significantly different (*P < 0.05, **P < 0.01, and ***P < 0.001) compared to control (0 µg/mL), as calculated using the two-tailed Mann-Whitney rank sum test. Source data are provided as a Source Data file.

The activity of purified plant-derived peptides, in parallel with synthetically generated peptides, was assessed against a panel of multi-drug resistant pathogens belonging to the group of clinically important ESKAPE [*E. coli* PI-7, methicillin-resistant *S. aureus* (MRSA) USA300*, K. pneumoniae, A. junii, P. aeruginosa*, and *E. faecalis*] bacterial pathogens using a standard killing assay in cation-adjusted Mueller-Hinton broth medium. All three plant-purified AMPs inhibited the growth of ESKAPE pathogens at concentrations between 6.25 µg/mL and 50 µg/mL (Fig. 4a–c, and Supplementary Figs. 3a–c, 4a, b, and Supplementary Tables 1, 2). Extracts obtained from wild type *N. benthamiana* didn’t exhibit any inhibition in the growth of ESKAPE pathogens (Supplementary Fig. 6a). Notably, purified AMP1 was slightly effective against *E. coli* PI-7 (50 µg/mL), a BSL-2 class pathogen and antibiotic-resistant strain^70^, against which colistin is the last resort antibiotic and drug of choice for treatment. In addition, our plant-purified peptides were highly effective against the community-acquired, BSL-2 class pathogen MRSA USA300 strain, which poses a threat to public health^71^ (MICs for AMP1, −2 and −3 = 25 µg/mL). We also tested the activity of all peptides against robust bacterial biofilm formation, which represents a notoriously drug-resistant state of microbes that is responsible for 65% of all infections, using crystal violet staining 24 h after treatment with the peptides. We achieved complete inhibition (>90%) of the Gram-positive bacterium MRSA USA300 biofilm with 25 µg/mL of AMP1 (Fig. 4d, e and Supplementary Fig. 5a), 12.5 µg/mL of AMP2 (Supplementary Figs. 3d, e, 5b), 25 µg/mL of AMP3 (Supplementary Fig. 4c) and >50% inhibition of *E. coli* PI-7 at 50 µg/mL of AMP1 (Fig. 4d, e and Supplementary Fig. 5a). The three peptides were also efficacious at preventing *K. pneumoniae* (Fig. 4d, f and Supplementary Figs. 3d, f, 4d, 5a, b), *A. junii* (Fig. 4d, f and Supplementary Figs. 3d, f, 4d, 5a, b), *E. faecalis* (Fig. 4d, f and Supplementary Figs. 3d, f, 4d, 5a, b), and *P. aeruginosa* (Fig. 4d, e and Supplementary Figs. 3d, e, 4c, 5a, b) biofilm formation, reflecting the widespread and robust antimicrobial activity of plant-produced peptides.

### AMP1 permeabilized the bacterial membrane, and killed cells

**Fig. 5:**
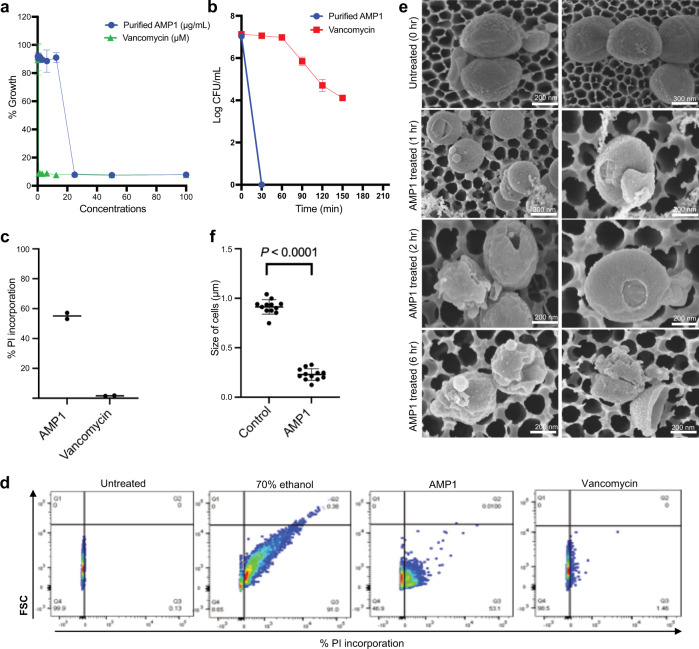
Plant-purified peptide AMP1 causes rapid membrane permeabilization and killing of MRSA USA300. **a** Antimicrobial activity (expressed as a minimal inhibitory concentration [MIC] of plant-purified AMP1 and vancomycin, evaluated against 10^6^ CFU/mL of MRSA USA300. MIC for pp AMP1 was ~25 µg/mL in tryptic soy broth; MIC for vancomycin was 0.7 µM (± SD, *n* = 3 independent experiments). **b, c** Killing kinetics (b) *n* = 3 independent experiments and influx of propidium iodide dye (c) as measured by flow cytometry. The controls used were as follows: x-axis, 0 = no antimicrobial activity; MRSA USA300 exposed to 70% ethanol is equivalent to 100% killing or influx of PI dye; and cell-wall biosynthesis inhibitor vancomycin (used at 2x MIC). **d** Percentage of PI-positive MRSA USA300 was calculated after addition of antimicrobial agents until their respective time points. **e** Scanning electron micrographs of MRSA USA300 treated with either PBS or 2x MIC of plant purified AMP1. **f** Mean cell width, as measured from SEM images by manually tracing the dimensions of individual cells. A standard two-tailed paired t test for analyzing the significance in the size of bacteria cells before (control) and after peptide treatment was applied. Data are means ± SD from three independent experiments. Source data are provided as a Source Data file.

Bacterial killing by synthetic AMP1 involves interaction with the bacterial outer membrane, followed by cytoplasmic membrane interaction/permeabilization^72^. To ascertain the mode of action of plant-purified AMP1, we determined its killing kinetics on the community-acquired multi-drug resistant clinical isolate MRSA USA300 strain in Ca-MHB (cation-adjusted Mueller-Hinton broth). We used vancomycin (last resort antibiotic that is effective against MRSA USA300) as a control that kills bacteria independently of membrane lysis. At a concentration of 2 x MIC, the plant-purified AMP1 completely killed an inoculum of 10^8^ colony-forming units (CFUs) of bacterial cells within 30-60 min of treatment (Fig. 5a, b), as observed previously for the native peptide. In contrast, the control antibiotic vancomycin required >2.5 h for bacterial killing, as expected.

We then looked for evidence for membrane permeabilization using flow cytometric analysis of influx of propidium iodide (PI, a normally impermeant dye) into bacterial cells at the time points mentioned above. The rate of PI incorporation due to plant-purified AMP1 was >50% (53.1%) as compared to control (Fig. 5d and Supplementary Fig. 9), suggesting membrane permeabilization while vancomycin, a cell-wall biosynthesis inhibitor, showed negligible PI accumulation (1.5%) (Fig. 5c, d). To verify the observed peptide-induced permeabilization of the plasma membrane, we employed scanning electron microscopy (SEM) imaging before and following treatment. Before the addition of the peptide or antibiotic, the cytoplasmic membrane of all cells was intact, but exposure to the peptide disrupted the bacterial membrane, as seen by the emergence of damage on the surface of several cells as well as shrinking of the cell size overall (Fig. 5e, f) which is in agreement with a previous study demonstrating similar effects using the synthetic peptide. These data indicate that plant-purified AMP1 displays similar activity as its synthetic counterpart by interacting with the bacterial membrane, leading to permeabilization and cell death.

### AMP1 synergized with azithromycin to exert bactericidal activity

**Fig. 6:**
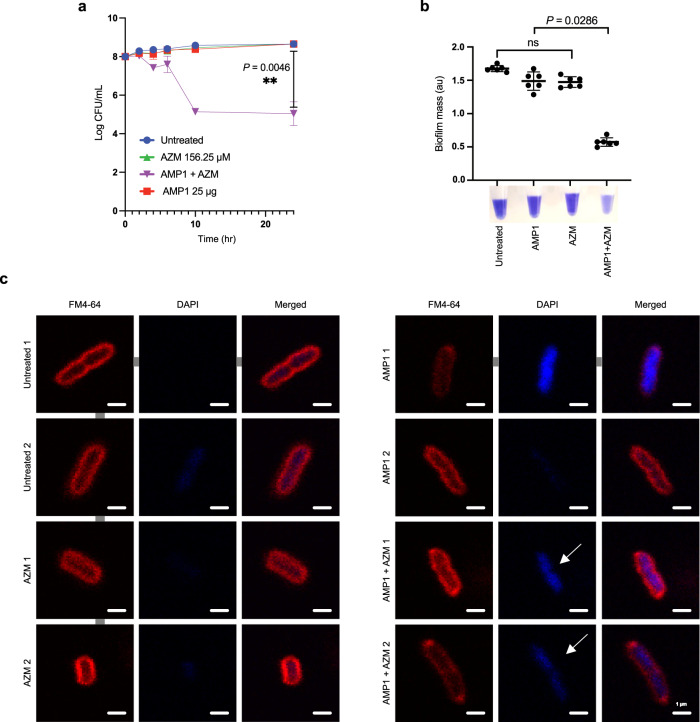
Purified peptides synergize with AZM by increasing the membrane permeability of carbapenem-resistant *E. coli* PI-7. **a** Time-kill curves and **b**, prevention of biofilm formation assay for drug-resistant *E. coli* PI-7. Nearly 10^8^ CFU/mL of *E. coli* PI-7 in the bacteriologic medium Ca-MHB were treated with AMP1 (25 µg/mL), AZM (156.25 µM) alone or in combination for 24 h. For time-dependent killing curve assay, CFU/mL was enumerated at 2, 4, 6, 10 and 24 h. In case of biofilm assay, 24 h treated culture was stained with 1% [v/v] crystal violet and results are expressed as biofilm mass, in arbitrary units (au). Data are means ± SD and represent the average of duplicates from 3 independent experiments. *P* = 0.0046, one-way ANOVA, Dunnett’s test and *P* = 0.0286, two-tailed Mann-Whitney rank sum test. **c** Overnight culture of *E. coli* PI-7 were diluted to 3 ×10^8^ CFU/mL in Ca-MHB and were treated with AMP1 (25 µg/mL), AZM (156.25 µM) alone or in combination for 2 h at 37 °C. After 2 h, culture was collected and stained with 1 μg/mL FM4-64 [(*N*-(3-Triethylammoniumpropyl)-4-(6-(4-(Diethylamino) Phenyl) Hexatrienyl) Pyridinium Dibromide)], 2 μg/mL DAPI (4’,6-diamidino-2-phenylindole). Stained cultures were centrifuged at 300× *g* for 30 s in a microcentrifuge and resuspended in approximately 5% original volume for fluorescence microscopy. Images were acquired using Leica Application Suite X version 3.5.5.19976 (www.leica-microsystems.com) and analyzed on Affinity designer Version 1.9.3. FM4-64 (red cell membrane stain), DAPI (blue DNA stain), and arrowheads indicate toroid-shaped nucleoids. Two independent microscopy experiments have been performed with similar results. Source data are provided as a Source Data file.

Colistin is usually a last resort antibiotic for carbapenem-resistant infections^73^, but its pharmacokinetics properties bring major risks for dose-dependent nephrotoxicity and uncertainties in optimal dosing^74^. To investigate whether plant-purified peptides could act synergistically with other antibiotics against which *E. coli* PI-7 has developed resistance, we screened for susceptible antibiotics using the standard broth-dilution method. *E. coli* PI-7 was highly resistant to gentamicin, kanamycin, ceftazidime, sulfamethoxazole, levofloxacin, ciprofloxacin (>350 µM), azithromycin (312.5 µM), but susceptible to colistin (20 µM) (Table 1). Next, we checked whether azithromycin could synergize with plant-purified AMP1, even in standard Ca-MHB, in which azithromycin alone has little or no activity. Indeed, we observed significant synergy at sub-MIC and pharmacologically attainable doses of both azithromycin + AMP1 (*P* = 0.0286; reduction by four orders of magnitude in CFUs/mL) against *E. coli* PI-7 (Fig. 6a). We confirmed this result with the marked prevention of biofilm formation when treated in combination (*P* = 0.046) compared to individual agents (Fig. 6b). Fluorescent microscopy showed a toroidal nucleoid morphology in azithromycin + AMP1 treated cells compared to either agent alone (Fig. 6c). We speculate that AMP1 markedly increased the membrane permeability, thus allowing for azithromycin to enter the cell more effectively and inhibit ribosomal protein synthesis.

**Table 1:** Antibiotic resistance profile of New Delhi metallo-β-lactamase-positive strain *E. coli* PI-7 isolated from sewage water

Antibiotics	MIC (μM)
Levofloxacin	>350
Ciprofloxacin	>350
Azithromycin	312.5
Ceftazidime	>350
Gentamicin	>350
Kanamycin	>350
Sulfamethoxazole	>350
Colistin	20

MIC- Mean inhibitory concentration expressed in micromolar concentration. Antibiotic resistance profile of *E. coli* PI-7 showing that the strain is resistant to antibiotics belonging to the fluoroquinolone class (MIC for levofloxacin and ciprofloxacin: >350 µM), macrolide (MIC for Azithromycin: 312.5 µM), cephalosporin (MIC for ceftazidime: >350 μM), aminoglycoside (MIC for gentamicin and kanamycin: >350 μM), sulfonamide (MIC for sulfamethoxazole: >350 μM) but highly susceptible to polymyxin (MIC for colistin: 20 μM). Source data are provided as a Source Data file.

### Techno-economic analysis for industrial-scale production of AMPs

Our established platform yielded a substantial amount of amidated AMPs; thus, we performed a techno-economic analysis to assess the feasibility of our developed module for industrial-scale production. Furthermore, a techno-economic analysis will establish the requirements, constraints, cost drivers, and capital cost estimation required to establish a large-scale AMP manufacturing facility. To this end, we performed simulation-based techno-economic analysis to predict the final cost of AMPs when produced on a large-scale. For the analysis, we have used the previously published base case scenario^75^, including the cost and installation of equipment, working capital, and the start-up cost, except for the cost of electricity, steam water, and labor charges which were estimated as per the local standard value (Supplementary Table 3). The base case scenario assumes to produce 91 batches a year with each upstream processing batch yielding 9,520 kg *N. benthamiana* plant FW containing 9.52 kg AMP, assuming an expression level of 1 g AMP per kg plant FW. The upstream processing steps (37% of total cost, Supplementary Fig. 7) include growing the plants, large-scale preparation of agrobacteria, vacuum-based infiltration, and post-infiltration incubation. The downstream processing steps (67% of total cost, Supplementary Fig. 8) involve harvesting leaves, homogenizing whole tissues, and extraction, retrieval, and chromatography-based purification of bulk AMPs. The SuperPro Designer® 13.0 software computed the cost of goods sold (COGS) at $74/g for amidated AMP (Supplementary Table 5). The final cost encompasses all materials (both raw and consumables), as well as the production costs for the additional chromatography step and protease purification from the *E. coli* strain that can secrete the target enzyme in the base case scenario. Additionally, the cost of each reagent used has been added in Supplementary Table 4, and the general COGS using different host chassis (*E. coli*^18,76^, mammalian cells^77^) is summarized in Supplementary Table 6.

## Discussion

The rapid emergence of drug-resistant bacteria, especially among members of the ESKAPE panel, is predicted to threaten up to 10 million lives each year by 2050^78^, underscoring the need for the judicious use of new strategies to develop antibiotics. AMPs constitute a promising alternative, since they possess potent antimicrobial and antibiofilm activity even against multi-drug resistant pathogens^79^. Despite decades of research and longstanding promise, no AMPs have been approved by the FDA, except cyclic lipopeptides and gramicidin S, although a few clinical trials have taken place or are underway^80^. The most significant obstacles impeding the clinical translation of peptides have been attributed to their poor serum stability, low efficacy due to shorter half-lives, and associated cost burden in manufacturing these expensive peptides^2^. To address these limitations, we designed a rapid, plant-based approach to produce amidated AMPs that are as efficacious as their synthetic counterparts and with the potential for scaled-up production.

Attempts to increase the production of cationic AMPs in plants have been met with lower yields than in *E. coli* cultures. In a previously reported transgenic *N. benthamiana* system, designed AMPs fused to the carrier protein β-glucuronidase (GUS) were not detectable by SDS-PAGE^43^. In another study, the stable transformation of rice (*Oryza sativa*) with a *cecropin A* construct that restricted peptide production to seed endosperm with no negative effect on seed physiology, but the yield was relatively low (0.5 − 6 μg/g seed tissue weight)^81^. In a completely different approach using the deconstructed virus system, magnICON, AMP protegrin-1 was sequestered to the apoplast of *N. tabacum* leaves; however, the yield was not reported in this case^82^. The low yield of these studies may reflect the incompatibility of the plant host chassis in the production of AMPs, although other factors may contribute as well, such as the presence of a cryptic splicing site in designed peptides leading to unfavorable RNA processing^83^, transcript formation and stability^84^, or aggregation of peptides due to their amphipathic nature^85^. These bottlenecks have been overcome in one study using a whole virus strategy, demonstrating a yield for cationic SP-1 peptide of 0.5 mg per 20 g of plant tissue using the tobacco mosaic virus system, where the designed AMP was fused to the viral coat protein^59^.

Here, we used *N. benthamiana* plants overexpressing rat *PAM1* to catalyze amidation *in planta*. These plants tolerated the stable integration of rat *PAM1* and exhibited no obvious morphological defects. In addition, *PAM1* plants were phenotypically normal and retained the ability to produce PAM1 at least up to the T4 generation, although they did produce far fewer seeds for an unknown reason. This effect on the reproductive system should, however, not constitute a major limitation for biotechnological applications. While efficient peptide amidation has been achieved so far in transgenic rabbits (*Oryctolagus cuniculus*)^86^, this approach requires a sizeable investment in centralized facilities for transgenesis, in contrast to plant transgenesis, which can be performed with minimal infrastructure. Besides, transgenic rabbits producing amidated peptides were reported to have precocious mammary development and reproductive problems^86^.

We reached production levels of 1.4 mg of peptide per 20 g of infiltrated leaf biomass. As a standard for comparison, 1 L of well-aerated *E. coli* yields 10-100 mg of AMPs^87^. Although not easily comparable, we consider that the amount of peptide reported in our study may represent an equivalent yield to an *E. coli* culture. No recoverable yield of cationic AMPs has been reported in plants thus far, as opposed to anionic AMPs that can accumulate to 4 − 113 mg per 200 g of plant tissue^46^. This discrepancy in charge-associated production has been attributed to the positive charge of peptides creating electrostatic attractions with the strong negatively charged plant membrane. Moreover, a meta-analysis study on all plant proteins in databases revealed that native AMPs belonging to the plant kingdom are less cationic than those from other taxa^46^.

Our data suggest that both the addition of the SUMO domain and the post-translational amidation catalyzed by PAM1 resulted in the stable accumulation of cationic AMPs in plants. The use of the SUMO domain has further beneficial effects for improved protein accumulation, presumably by increasing protein stability and solubility, thus facilitating AMP purification without adding high concentrations of detergents. A recent publication reported the production of AMPs in plastids using a similar approach involving AMPs fused to an intact SUMO domain^88^. However, compared to our simple expression module for AMP production, which can be applied to many plant species, plastid engineering is sophisticated and suffers from technical hurdles limiting its application. Also, compared to plastid-expressed AMPs associated growth defects, our engineered AMPs constructs expression did not inhibit plant growth. More importantly, our study provides a simple production strategy to obtain amidated AMPs, and our platform is amenable to other expression strategies, which will facilitate scale-up production of clinical grade AMPs.

Previous studies have demonstrated that the synthetic AMPs used in our study are active against both Gram-positive and Gram-negative bacteria. In our work, we demonstrated that plant-produced AMPs were similarly active against several ESKAPE pathogens, indicating that their biological activity is preserved. Moreover, the plant-produced AMPs described here caused membrane permeabilization that likely contributed to the killing of the bacterial pathogens. A particularly interesting finding from our antibiofilm assay was that plant-produced peptides were highly potent in preventing biofilm formation against MRSA USA300, in agreement with results previously obtained for synthetically produced forms of these peptides^42^. Since biofilms represent a highly drug resistant growth state of bacteria, this observation warrants further clinical validation for the management of biofilm-associated infections. In addition, plant-produced peptides exhibited marked synergism with azithromycin in curtailing the growth rate of carbapenem-resistant strain *E. coli* PI-7, potentially adding another antibiotic to clinical management for this strain for which colistin is the last resort drug.

Our optimized protocol yielded a substantial amount of pure amidated AMPs (> 90%), and this prompted us further to compute the scalability of this process for industrial-scale production of AMPs. The techno-economic analysis simulation estimated the total cost of goods sold (COGS) at $74/g for plant-based production of AMPs. This cost is quite competitive considering that chemical synthesis of the same peptide was priced at $95.29/mg (based on a price quote from a commercial company) and compared against the COGS of *E. coli* produced cationic peptides produced in batches which ranges from $44.5-$268.16/mg^18^. Furthermore, protein production in mammalian cells generally represents an expensive proposition with the associated cost usually priced at > $1450/g, and to produce similar titer with plant-based system costs < $100/g, representing an overall > 50% reduction in cost^75^. Hence, even though multiple chromatography steps can be involved, plant-based AMPs production still requires significantly less capital investment and lower cost of goods compared to *E. coli* fermentation and mammalian cultures. Thus, taking into consideration the techno-economic analysis, our manufacturing platform provides a sustainable approach towards the production of peptides by incorporating the green chemistry route and avoiding the use of hazardous materials. Moreover, this platform is amenable to peptides of varying lengths, which may be challenging to produce via SPPS.

Our study opens avenues for the future prospect of plants as a biomanufacturing chassis and production platform for biologically active peptides irrespective of their charge, which had previously proven difficult. Moreover, based on the relaxed specificity offered by the PAM enzyme for the penultimate amino acid that is amidated, as well as the potential to scale up, we envision that our efficient synthetic engineering approach could lead to improved plant-based industrial-scale production of amidated therapeutics.

## Methods

### Construction of synthetic glycine-extended AMPs for *in planta* expression

All synthetic antimicrobial gene cassettes comprising sequences encoding the streptavidin-tag II, 3x HA epitope, mutated bdSUMO^Eu1^ module fused to the N terminus of each AMP and the terminal glycine residues were ordered as gBlocks gene fragments (Supplementary Data 1, IDT, Leuven, Belgium). The engineered SUMO^Eu1^ module was previously shown to resist proteolytic cleavage by endogenous deSUMOylases in eukaryotic cell lysates, facilitating the isolation of protein complexes from eukaryotic extracts^53^. The sequences of *AMP* genes were codon-optimized to increase the translational efficiency in the production host *Nicotiana benthamiana*. Each synthetic gBlocks template was PCR amplified with primers that added AgeI and XhoI restriction sites to the 5’ and 3’ ends of the PCR product, respectively, for subsequent cloning into the AgeI/XhoI-digested *Cowpea mosaic virus*-based vector pEAQ-HT (Leaf Expression Systems, Norwich, UK). The constructs were verified by Sanger sequencing using a forward primer complementary to the vector backbone and a reverse primer complementary to the 3’ end sequence of the *AMP* gene cassette. All oligonucleotides were purchased from Integrated DNA Technologies (IDT, Leuven, Belgium) and were HPLC-purified by the manufacturer. Sequences of the oligonucleotides are listed in Supplementary Data 2.

### Cloning of the rat *PAM* gene

Plasmids encoding the rat variants of PAM enzymes (PAM 1, 2 & 3) were kindly provided by Prof. Betty Eipper, University of Connecticut Health Center, USA. Using PCR, the coding sequence encoding the bifunctional PAM enzymes encompassing the peptidylglycine α-hydroxylating monooxygenase (PHM) domain, peptidyl-α-hydroxylglycine α-amidating lyase (PHL) domain, the transmembrane domain, and the cytosolic region were amplified from plasmid DNA. The *PAM2* variant lacks exon 16 located adjacent to the sequence encoding the protease-sensitive region separating the PHM and PHL domain, whereas *PAM3* variant lacks the sequence encoding transmembrane domain. Public database used for rat PAM enzyme sequence include UniProt (https://www.uniprot.org/uniprotkb/A0A8I5ZMR1/entry). To facilitate directional cloning into the intermediate vector pENTR™ D-TOPO® (Invitrogen), the forward primer was preceded by the four nucleotides CACC. The reverse primer contained unique restriction sites for HindIII and XbaI to ligate the annealed HA primers with overhanging sticky ends complimentary to HindIII/XbaI. The subcloned vectors containing the *PAM-HA* construct were verified by Sanger sequencing using overlapping primers. Next, the inserts were recombined into the plant transformation vector pK2GW7 using Gateway cloning to drive the expression of *PAM* genes under the control of the constitutively active cauliflower mosaic virus (CaMV) 35 S promoter.

### Generation of transgenic *N. benthamiana* plants overexpressing *PAM*

The pK2GW7 binary vectors containing the various *PAM* genes generated above were introduced into Agrobacterium (*Agrobacterium tumefaciens*) strain GV3101 by electroporation. Stable Agrobacterium-mediated leaf disc transformation was performed according to a standard protocol^89^. Briefly, 2-week-old *Nicotiana benthamiana* leaf explants infiltrated with *Agrobacterium* containing *PAM genes* in MES buffer (10 mM 2-[N-morpholino]-ethanesulfonic acid, pH 5.6, 10 mM MgCl_2_, 100 μM acetosyringone) were grown onto solid MS media (4.4 g/L Murashige and Skoog basal salts [MS], 30 g/L sucrose) in a growth chamber with the temperature set to 28 °C. After 2 days, the explants bearing the integrated transgene were selected and regenerated onto media (4.4 g/L MS salts, 1 mg/L 6-benzylaminopurine, 0.1 mg/L 1-naphthaleneacetic acid, 30 g/L sucrose, 50 mg/L kanamycin, 200 mg/L timentin, pH 5.8) in a growth chamber with the temperature set to 25 °C and a 13-h light/11-h dark regime. Following growth hormones-mediated shoots induction which take at least 3-4 weeks, the shoots were excised and transferred for rooting onto media (2.2 g/L MS salts, 50 mg/L kanamycin). All the reagents were purchased from Sigma-Aldrich. Finally, kanamycin-resistant lines forming proper roots (2-3 weeks) were acclimatized to the soil in greenhouse under plastic domes with the temperature set to ~28–30 °C for continued growth until maturity. Transgenic plants were propagated until the homozygous T_4_ generation and were screened using immunoblot for accumulation of the PAM protein.

### Purification of SENP^EuH^ protease

A plasmid encoding the *Brachypodium distachyon* mutated protease His-TEV-SENP^EuH^ was purchased from Addgene (plasmid number 149689) and transformed into *E. coli* BL21 (DE3) pLysS cells (New England Biolabs Inc., Hitchin, England). SENP1^EuH^ protease was produced by growing bacteria into 2 L of Terrific broth (IBI Scientific) containing kanamycin. Cells were grown at 37 °C until reaching an OD600 of 0.5-0.7; protein production was induced by the addition of isopropyl-β-D-thiogalactopyranoside (IPTG) at a final concentration of 0.3 mM. The cells were grown at 18 °C for 19 h, harvested by centrifugation at 5,500 x g for 15 min at 4 °C, then resuspended in ice-chilled lysis buffer (50 mM Tris-HCl pH 7.5, 300 mM NaCl, 4.5 mM MgCl_2_, 5% [v/v] glycerol, 20 mM imidazole, 100 mM PMSF and complete EDTA-free protease inhibitor cocktail tablet/50 mL [Roche, UK]). The cells were subjected to lysis using lysozyme (Sigma) at a concentration of 1 mg/mL on ice for 1 h, followed by mechanical disruption using sonication (Qsonica Q700). Cell debris were then removed by centrifugation at 10,000 x g for 40 min at 4 °C and the decanted supernatant was passed through a Nalgene disposable bottle top filter with a 0.45-μm membrane (Thermo Fisher Scientific, USA). The filtered supernatant was loaded onto a 5-mL HisTrap^TM^ HP column (GE Healthcare Biosciences) pre-equilibrated with buffer A (50 mM Tris-HCl pH 7.5, 500 mM NaCl, 20 mM imidazole, 5% [v/v] glycerol) using an ÄKTA pure instrument (UNICORN 6.3, GE Healthcare Biosciences). The column was extensively washed with ten column volumes (CVs) of buffer A and the bound protease was eluted in ten CV fractions against buffer B (50 mM Tris pH 7.5, 500 mM NaCl, 500 mM imidazole, 5% [v/v] glycerol). The fractions containing the SENP^EuH^ protease was analyzed using SDS-PAGE, pooled, and dialyzed overnight in Snakeskin-pleated dialysis tubing (Thermo Fisher Scientific, USA) against dialysis buffer (25 mM Tris pH 7.5, 100 mM NaCl, 1 mM DTT, 10% [v/v] glycerol). The dialyzed sample was concentrated to 1 mL using centrifugal filters with a membrane NMWL of 10-kDa (Millipore, USA). The concentrated protein was then loaded onto a HiLoad 16/600 Superdex 200 pg gel filtration column (GE Healthcare Biosciences) equilibrated with storage buffer (25 mM Tris pH 7.5, 100 mM NaCl, 1 mM DTT, 10% [v/v] glycerol). Fractions containing the protease were pooled, flash-frozen in liquid nitrogen and stored at −80 °C until use.

### Large-scale purification of peptides

Leaves infiltrated with each *AMP* construct were harvested 6 days post-infiltration and ground in liquid nitrogen to a fine powder with pre-cooled mortars and pestles. Total proteins were extracted from the leaf powder by the addition of 2–3 × (w/v) ice-cold extraction buffer (100 mM Tris-HCl pH 8.0, 150 mM NaCl, 1 mM EDTA, 3 mM DTT, 4% [w/v] polyvinylpolypyrrolidone [PVPP], 0.1% [v/v] Triton X-100, 100 mM PMSF and Complete EDTA-free protease inhibitor cocktail tablet/30 mL [Roche, UK]), followed by mechanical disruption using sonication at 30% amplitude. Since the phenol adsorbent polymer PVPP is highly insoluble in polar solvents, it was directly added to the ground leaf powder. The slurry was completely squeezed through 2-3 layers of Miracloth, clarified by centrifugation at 10,000 x g for 1 h at 4 °C and filtered through a Nalgene disposable bottle top filter with a 0.45-µm membrane (Thermo Fisher Scientific, USA). The filtered supernatant was applied to 5 mL of *Strep*-Tactin Superflow resin (Qiagen, Hilden, Germany) in gravity flow Econo-columns® (BioRad), incubated for 2 hr at 4 °C with gentle rotation, followed by resin washes with buffer (100 mM Tris-HCl pH 8.0, 150 mM NaCl, 1 mM EDTA, 3 mM DTT) to remove loosely bound proteins. After washing the resin, it was immediately resuspended in SUMO digestion buffer (45 mM Tris-HCl pH 7.5, 2 mM MgCl_2_, 250 mM NaCl, 10 mM DTT, 0.1% [v/v] NP-40). Recombinant AMPs were released under native form by overnight cleavage with 17 µg of purified SENP^EuH^ protease in the presence of 1 M Urea at 4 °C under gentle rotation. Urea was added to the protease reaction buffer for precise cleavage of the peptide and to prevent any nonspecific activity. Cleaved AMPs were collected and loaded onto a 5-mL HisTrap^TM^ HP column (GE Healthcare Biosciences) using buffer A (50 mM Tris-HCl pH 7.5, 300 mM NaCl, 20 mM imidazole) via ÄKTA pure (GE Healthcare Biosciences) to remove His-tagged SENP^EuH^ protease. The wavelengths used to monitor the cleaved peptides were 215 nm (peptide bond absorbs light at 215 nm) and 280 nm since all three prototypical AMPs have an aromatic-side chain of tryptophans that absorbs light in the UV range of 250–290 nm, thereby providing a convenient means for peptide detection. Flow-through fractions that were devoid of protease but contained the native AMPs were collected and immediately freeze-dried in a lyophilizer to concentrate the fractions. The lyophilized extract was then resuspended in size-exclusion chromatography (SEC) buffer (5% [v/v] HPLC-grade CH_3_CN, 0.01 M HCl and 150 mM NaCl) and centrifuged at 10,000 g maintained at room temperature for 10 min. The cleared supernatant was injected in a 1.5-mL loop, loaded onto a SEC-buffer pre-equilibrated Superdex 30 Increase 3.2/300 (GE Healthcare Biosciences) with a flow rate of 0.01 mL/min, monitored at 215 nm/280 nm and eluted in SEC buffer. The peak fractions were analyzed on 18% Tricine-SDS-PAGE gels, flash-frozen in liquid nitrogen, and stored at –80 °C until further processing. Peptide fractions were further desalted and separated using reverse-phase chromatography. The semi-preparative column used was 9.4 ×250 mm ZORBAX RX-C8 with particle size of 5 μm (Agilent Technologies, USA), fractions containing peptides were loaded onto the column using the 1-mL loop present in the 1260 Infinity HPLC system (OpenLAB CDS ChemStation C.01.07 SR2, Agilent Technologies, USA) and eluted using linear gradient from 5% (v/v) CH3CN/0.01 M HCl to 80% (v/v) CH3CN/0.01 M HCl at a flow rate of 1 mL/min. Fractions absorbing at 215 nm/280 nm were then collected, subjected to freeze-drying and stored at –20 °C until use.

### ESI-TOF-MS of peptides

Mass identification of peptides was carried using a MicroTOF-Q spectrometer (Bruker Daltonics, Inc, Germany). The machine was calibrated in positive ionization mode using 1% (v/v) formic acid in acetonitrile/HPLC-grade water solution (CH_3_CN/H_2_O, 50/50, v/v). Dried peptide samples were dissolved in solvent containing 50% (v/v) CH_3_CN and 1% (v/v) formic acid and injected into the ESI source using a stainless-steel needle syringe at a flow rate of 10 µL/min. Data were acquired by the TOF analyzer (Compass for otofSeries 1.7 Version 3.4, Bruker Daltonics GmbH) at a rate of 1 acquisition/sec from m/z 200 to m/z 2000. The optimized voltage was set to +3 kV for the capillary and dry nitrogen gas heated to 150 °C was used for better nebulization. Data were acquired and processed with the Compass DataAnalysis software (Bruker Compass DataAnalysis 4.2 SR2, Bruker Daltonics GmbH).

### Antibiotics and synthetic antimicrobial peptides

For in vitro studies, azithromycin (Cat. no. PZ0007), colistin sulfate (Cat. no. C4461), meropenem (Cat. no. PHR1772), ceftazidime (Cat. no. C3809), vancomycin sulphate (Cat. no. 861987), sulfamethoxazole (Cat. no. S7507), gentamicin (Cat. no. G1914), kanamycin (Cat. no. BP861), levofloxacin (Cat. no. 28266), ciprofloxacin (Cat. no. 17850) were purchased from Sigma. The stock solutions of antibiotics were prepared in molecular biology grade 1 x phosphate-buffered saline (PBS) (Corning Inc., Corning NY, USA). In case of azithromycin, trace amounts of glacial acetic acid was added for complete solubility. Synthetic QCed peptides AMP1, AMP2 were kindly provided by Prof. Robert Hancock (University of British Columbia, Canada). Peptides were dissolved in endotoxin-free sterile water (Corning Inc., Corning NY, USA) containing 0.025% (v/v) acetic acid and 0.1% [w/v] bovine serum albumin (BSA) for in vitro experiments.

### Bacterial strains and media

The pathogenic strains used in this study were carbapenem-resistant *Escherichia coli* PI-7 (a New Delhi metallo-β-lactamase-positive strain previously isolated from municipal wastewater in Saudi Arabia), methicillin-resistant *Staphylococcus aureus* USA300, extended-spectrum β-lactamase-producing *Klebsiella pneumoniae* ATCC 700603, *Acinetobacter Junii* DSMZ 14968, *Pseudomonas aeruginosa* ATCC 9027, *Enterobacter faecalis* ATCC 29212. Pathogenic *Escherichia coli* PI-7 was grown in LB broth containing 8 μg/mL meropenem, methicillin-resistant *Staphylococcus aureus* USA300 was grown in tryptic soy broth (TSB; Difco, Detroit) containing 10 μg/mL chloramphenicol, while all remaining strains were grown in LB broth without any antibiotic added.

### Minimal inhibitory concentration (MIC) assay

The MIC values were determined using broth microdilutions in accordance to the Clinical Laboratory Standards Institute (CLSI) guidelines using cation-adjusted Mueller-Hinton broth (Ca-MHB) with minor modifications^90^. Briefly, bacteria were grown overnight in the appropriate media at 37 °C with shaking. The overnight culture was washed with PBS and centrifuged at 3220 ×g at room temperature for 10 minutes with a final resuspension in PBS to an OD600 = 0.50 approximating 10^8^ CFU/mL. Bacterial stocks were diluted to an inoculum of 10^6^ CFU/mL in Ca-MHB. Ninety microliters of this suspension were added to each well of 96-well round bottom plates (Costar) along with 10 µL of diluted antibiotics/peptides at varying concentrations. The 96-well plates were wrapped in paraffin and placed in a shaking incubator at 37 °C. The OD600 readings were taken after 24 h using a TECAN Infinite 200 PRO series (Tecan i-control 2; 2.0.10.0, Austria, GmbH). The MIC was considered as the lowest concentration of peptide that completely inhibited the visible growth of bacteria after 24 h of incubation of the plates at 37 °C. Data are presented as two independent experiments performed in duplicates.

### Immunoblot analysis

All plant expression constructs carried the sequence encoding a triple N-terminal hemagglutinin (HA)-epitope tag to analyze production abundance by immunoblot. Leaves were harvested post-infiltration and total protein was extracted from 100 mg of sample using extraction buffer (100 mM Tris-HCl pH 8, 5 mM EDTA, 150 mM NaCl, 10 mM DTT, 0.5% [v/v] Triton X-100 along with protease inhibitor cocktails consisting of 1 mM PMSF, 15 µg/mL leupeptin, 1 µg/mL aprotinin, 1 µg/mL pepstatin, 5 µg/mL antipain, 5 µg/mL chymostatin, 2 mM Na_2_VO_3_, 2 mM NaF, 10 µM MG132). Low molecular-weight peptides (<20 kDa) were separated on 18% Tricine-SDS-PAGE gels. Proteins > 20 kDa were resolved on 4-20% Tris-HEPES gels (Thermo Fisher Scientific, USA). The separated proteins were transferred to a polyvinylidene difluoride membrane with a pore size of 0.45 µm (Amersham Hybond-P; GE Healthcare Life Sciences). The membranes were blocked with 5% (w/v) BSA (Sigma), incubated overnight with primary antibodies rat anti-HA (1:1000, Sigma, clone name: 3F10), anti-GFP (1:1500, ab6556, polyclonal) and subsequently with respective HRP-conjugated secondary antibodies goat anti-rat IgG (1:4000, Sigma), goat anti-rabbit IgG (1:2000, ab205718). Immunoblotting bands were visualized using enhanced chemiluminescence reagent (ECL kit; Amersham Pharmacia Biotech) and blot images were acquired using a ImageQuant LAS 4000 (Version 1.0, GE Healthcare Biosciences). In case of SDS-PAGE, images were acquired using a ChemiDoc MP system (Image lab Version 6.0.1, BioRad).

### Membrane permeabilization assay

A mid-logarithmic growth-phase culture was diluted to 1 × 10^8^ CFU/mL in Ca-MHB and was exposed to antimicrobial agents for the estimated time as evaluated in time-kill kinetic assay for each respective agent. Twenty microliters of propidium iodide (PI, Molecular Probes, Invitrogen) with a final concentration of 1 µg/mL were then added to the cells and incubated in the dark for 30 min. The percent influx of PI stain was then analyzed using a BD LSRFortessa™ Cell Analyzer (BD FACSDiva Software, Version 6.2, BD Biosciences, San Jose, CA, USA) and calculated using FlowJo 10.6.2 software (BD Biosciences).

### Cell cultured and live/dead staining

Human embryonic kidney 293 (HEK-293) cells (Thermo Fisher Scientific, Cat. no. 51-0035) were cultured in 75 T flasks and incubated in a humidified incubator maintained at 37 °C with 5% (v/v) CO_2_ using DMEM/high-glucose medium supplemented with Glutamax, 10% (v/v) fetal bovine serum (FBS), and 1% (w/v) penicillin/streptomycin (GIBCO, Thermo Fisher Scientific, USA). The culture medium was replaced every 2 days until the cells reached 80% confluency. Cells were sub-cultured and seeded at a density of 1×10^4^ cells per well in 96 well-plates. Then, 50 µg/mL of each peptide (maximum reported MIC) was added to the cells. After 2 days of incubation, 2 mM of calcein AM and 4 mM ethidium homodimer-1 (LIVE/DEAD® Viability/Cytotoxicity Kit, Life Technologies^TM^) was added to the wells and incubated for 40 min in the dark. Before imaging, the staining solution was removed, and fresh PBS was added. Stained cells were imaged under an inverted confocal microscope (Zeiss Microscope, Germany).

### Proliferation assay

A CellTiter-Glo® luminescent 3D cell viability assay was used to determine proliferation of cells according to the amount of ATP produced as an indicator of cellular metabolic activity. About 1×10^4^ of cells were seeded per well of a 96-well plate. Then, 50 µg/mL of each peptide was added to the cells. After the incubation time, the kit was equilibrated at room temperature for approximately 30 min. CellTiter-Glo® Reagent equal to the volume of cell culture medium present in each well was added. The contents were mixed for 5 min and then incubated for 30 min. After incubation, the luminescence was recorded using a plate reader (PHERAstar FS, Germany).

### Cytoskeleton staining

Immunostaining was performed after the incubation of each peptide with cells for 84 hr as described previously^91^. Briefly, cells were fixed with 4% (w/v) paraformaldehyde solution for 30 min and incubated in cold cytoskeleton buffer (3 mM MgCl_2_, 300 mM sucrose and 0.5% [v/v] Triton X-100 in PBS) for 5 min for permeabilization. The permeabilized cells were incubated in blocking buffer solution (5% [v/v] FBS, 0.1% [v.v] Tween-20, and 0.02% [w/v] sodium azide in PBS) for 30 min at 37 °C. Then, F-Actin, rhodamine-phalloidin (1:300) was added to the cells that were then incubated at room temperature in the dark for 1 h, followed by washing three times with 1X PBS. Further, the cells were incubated in DAPI (1:2,000) in water for five min to counterstain the nucleus before the DAPI solution was removed by washing with 1X PBS. The stained cells were observed and imaged using a laser scanning confocal microscope (Leica Application Suite X, Leica Stellaris Confocal Microscope, Germany).

### Prevention of biofilm formation

A mid-logarithmic growth-phase culture was diluted in BM2 medium (62 mM potassium phosphate buffer, pH 7, 7 mM (NH_4_)_2_SO_4_, 2 mM MgSO_4_, 10 μM FeSO_4_ and 0.4% [w/v] glucose) to 1×10^8^ CFU/mL and 90 µL of this suspension was seeded in polypropylene microtiter plates (Corning Inc., Corning NY, USA). Bacterial cells were then exposed to varying concentration of AMPs (100 µg/mL to 1.56 µg/mL) and grown overnight at 37 °C in a humidified atmosphere. As an untreated control, bacteria were exposed to BM2 medium without any peptide. After 24 h of incubation, planktonic bacterial growth was aspirated out, biofilms were fixed with 100% methanol for 15 min, washed with PBS and finally air-dried. Dried biofilms were stained with 1% (w/v) crystal violet (Sigma) for 30 min, washed with PBS, and solubilized in 95% (v/v) ethanol for 1 h. The optical density at 595 nm was recorded using TECAN Infinite 200 PRO series (Tecan i-control 2; 2.0.10.0, Austria, GmbH) as a measure of biofilm mass.

### Scanning electron microscopy (SEM)

Untreated bacterial cells were prepared in Ca-MHB and fixed overnight with modified Karnovsky’s fixative (2.5% [w/v] glutaraldehyde and 2% [w/v] paraformaldehyde in 0.1 M sodium cacodylate buffer, pH 7.35) at 4 °C. For peptide-treated cells, suspended cells were filtered using a commercial 50-mL vacuum filter with a 0.22-μm pore-size membrane (Corning Inc., Corning NY, USA) and directly used for fixation. Thereafter, specimens were post-fixed with 1.5% (w/v) potassium ferrocyanide, and 1% (w/v) osmium tetroxide prepared in 0.1 M sodium cacodylate buffer, dehydrated through a graded ethanol series, dried using a critical point dryer (CPD300, Leica, Germany) and sputter-coated with a 10-nm thick platinum layer. All specimens were imaged using a FEI Nova Nano 630 SEM (SmartSEM Version 6.09, Serial Number Merlin-61-95, Oregon, USA) equipped with an Everhart-Thornley detector (ETD) and through a lens detector (TLD) operating at 3 kV.

### Techno-economic analysis for industrial-scale production of AMPs

We performed a techno-economic analysis to ensure the feasibility of our developed module for industrial-scale production to establish the requirements, constraints, cost drivers, and capital cost estimation required for a large-scale AMP manufacturing facility. Plant-based AMP production and purification was modelled on a previously published base case scenario^75^ using the SuperPro Designer® 13.0 software (https://www.intelligen.com; accessed from December 2nd to 15th, 2022) considering the upstream processing batch yields 9,520 kg *N. benthamiana* plant FW containing 9.52 kg AMP, with an expression level of 1 g AMP per kg plant FW. The model proposed vacuum-based infiltration of *N. benthamiana*, wherein plants were infiltrated transiently using vacuum in batches. Seed germination efficiency was considered to be >95%, with an estimated cost of $9.50/g seed. The overall COGS was calculated considering all materials (raw and consumables) including the production costs (utilities, facility-dependent costs, waste disposal and labor) divided by the product output. Electricity, labor, hot and cold-water charges were estimated as per the Saudi Arabia local standards value. All currency is listed in USD.

### Statistical analysis

Where applicable, data are shown as means ± SD with error bars. Two-sample comparisons were made using a Mann-Whitney rank sum test. For all tests, *P* < 0.05 was considered significant.

### Reporting summary

Further information on research design is available in the Nature Portfolio Reporting Summary linked to this article.

## Supplementary information

Supplementary Information

Description of Additional Supplementary Files

Supplementary Data 1

Supplementary Data 2

Reporting Summary

## Data Availability

A reporting summary for this Article is available as a Supplementary Information file. Data supporting the findings of this work are available within the paper and its Supplementary Information files. Source data are provided with this paper.

## Source data

Source Data
