# Supplementary material for: Efficient in planta production of amidated antimicrobial peptides that are active against drug-resistant ESKAPE pathogens
Source: Nat Commun. 2023 Mar 16;14:1464. doi: 10.1038/s41467-023-37003-z (PMC10020429; doi:10.1038/s41467-023-37003-z)
Supplement: Supplementary file 1 — Supplementary Information [file 41467_2023_37003_MOESM1_ESM.pdf]

Efficient *in planta* production of amidated antimicrobial peptides that are active against drug-resistant ESKAPE pathogens

Chaudhary *et al*.

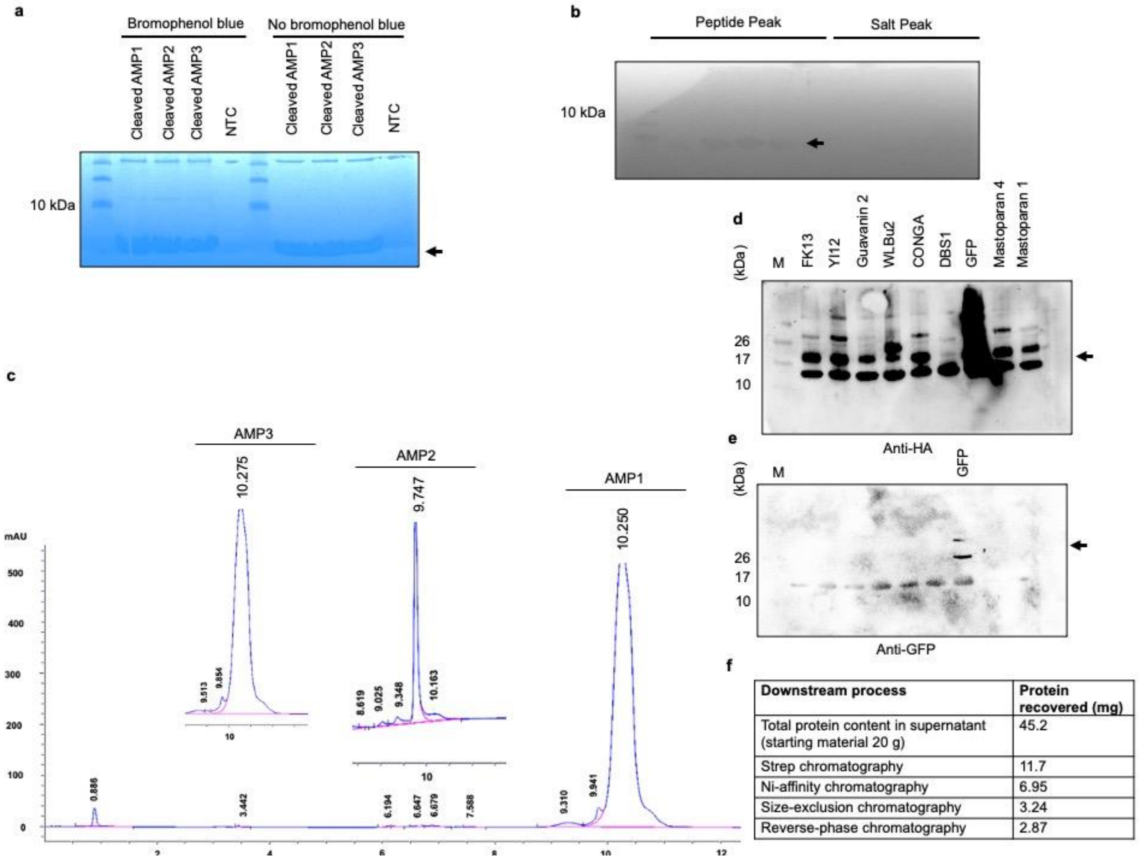

Downstream process	Protein recovered (mg)
Total protein content in supernatant (starting material 20 g)	45.2
Strep chromatography	11.7
Ni-affinity chromatography	6.95
Size-exclusion chromatography	3.24
Reverse-phase chromatography	2.87

Supplementary Fig. 1. Establishment of a SynBio chassis for *in planta* expression of AMPs.

a, Cleaved peptide fractions were run on a 18% Tricine-SDS-PAGE with gel loading dye containing bromophenol blue or without bromophenol blue. Native low molecular-weight peptides migrated to the bottom of the gel and the 32.6-kDa protease band can be seen at the top of the gel. Protein extracts obtained from plants infiltrated with empty pEAQ-HT vector was used as negative control. **b,** Cleaved AMP fractions were purified using size exclusion chromatography (SEC) in buffer containing 150 mM NaCl, 5% (v/v) CH_3CN, 0.01 M HCl and analyzed on 18% Tricine-SDS-PAGE gel. Two independent Tricine-SDS-PAGE have been performed with similar results. **c,** Pooled SEC fractions were run on a 9.4 x 250 mm ZORBAX RX-C8 column and monitored at the two wavelengths 215 nm and 280 nm. **d,** Abundance of Strep-II tag-captured SUMO-fused FK13, YI12, Guavanin 2, WLBU2, CONGA, DBS1, GFP, Mastoparan 4,1 as detected with a monoclonal anti-HA after transient infiltration in transgenic *N. benthamiana* expressing *PAM1*. **e,** The immunoblot was stripped and reprobed with a monoclonal anti-GFP antibody to test HA-tagged SUMO-GFP accumulation. Two independent blots have been performed with similar results. **f**, Table showing concentration of proteins recovered at each step in the downstream process for *in planta* peptide purification. The black arrowheads indicate to the corresponding proteins and peptides. Source data are provided as a Source Data file.

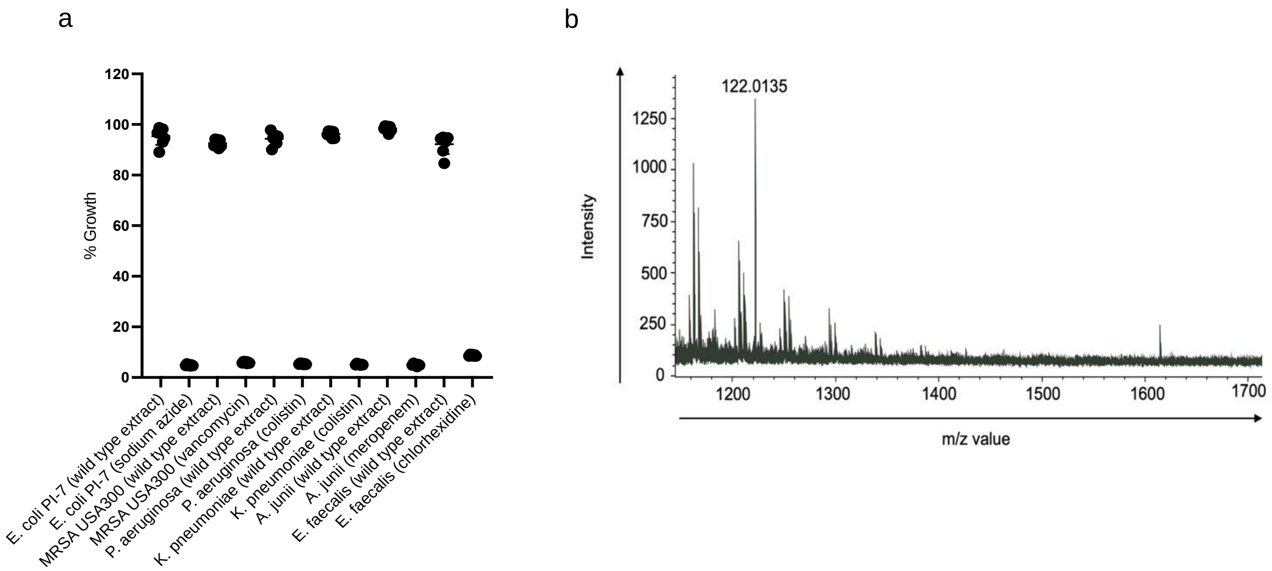

Supplementary Fig. 2. Wild type extract activity against ESKAPE pathogens and their characterization using ESI-MS.

a, Protein extract obtained from wild type *N. benthamiana* were subjected to the same purification procedure and dissolved in the peptide buffer containing 0.025% (v/v) acetic acid and 0.1% [w/v] bovine serum albumin (BSA). The activity was determined by incubating with ESKAPE pathogens for 24 h followed by absorbance readings at OD600. As a positive control, *E. coli* PI-7 (100 µg/mL sodium azide), MRSA USA300 (40 µM colistin), *P. aeruginosa* (40 µM colistin), *K. pneumoniae* (40 µM colistin), *A. junii* (16 µg/mL meropenem), *E. faecalis* (2% chlorhexidine prepared in 70% ethanol) were used. Data are means ± SEM of two independent experiments. **b,** Mass analysis of wild type *N. benthamiana* extract using ESI-MS. The y-axis shows the signal intensity, and the x-axis displays the m/z value. Source data are provided as a Source Data file.

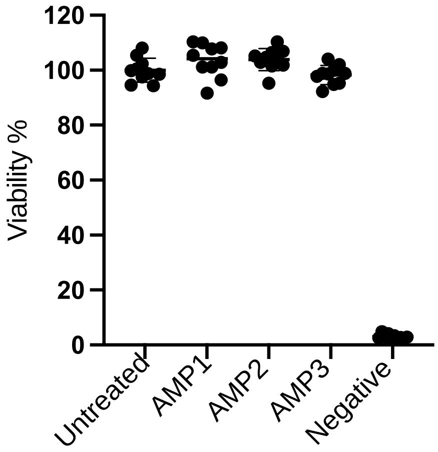

Supplementary Fig. 3. Plant-purified peptides display low toxicity in mammalian cells.

Bar graph illustration of metabolic index of both peptides treated (50 µg/mL) and untreated HEK293 cells as measured by estimating the cellular ATP levels using luciferase-based CellTiter-Glo® reagent. Cell viability was quantified as the ratio of ATP levels-dependent luminescence in treated / untreated cells expressed as percentage (± SD, *n* = 3 independent biological replicates). Source data are provided as a Source Data file.

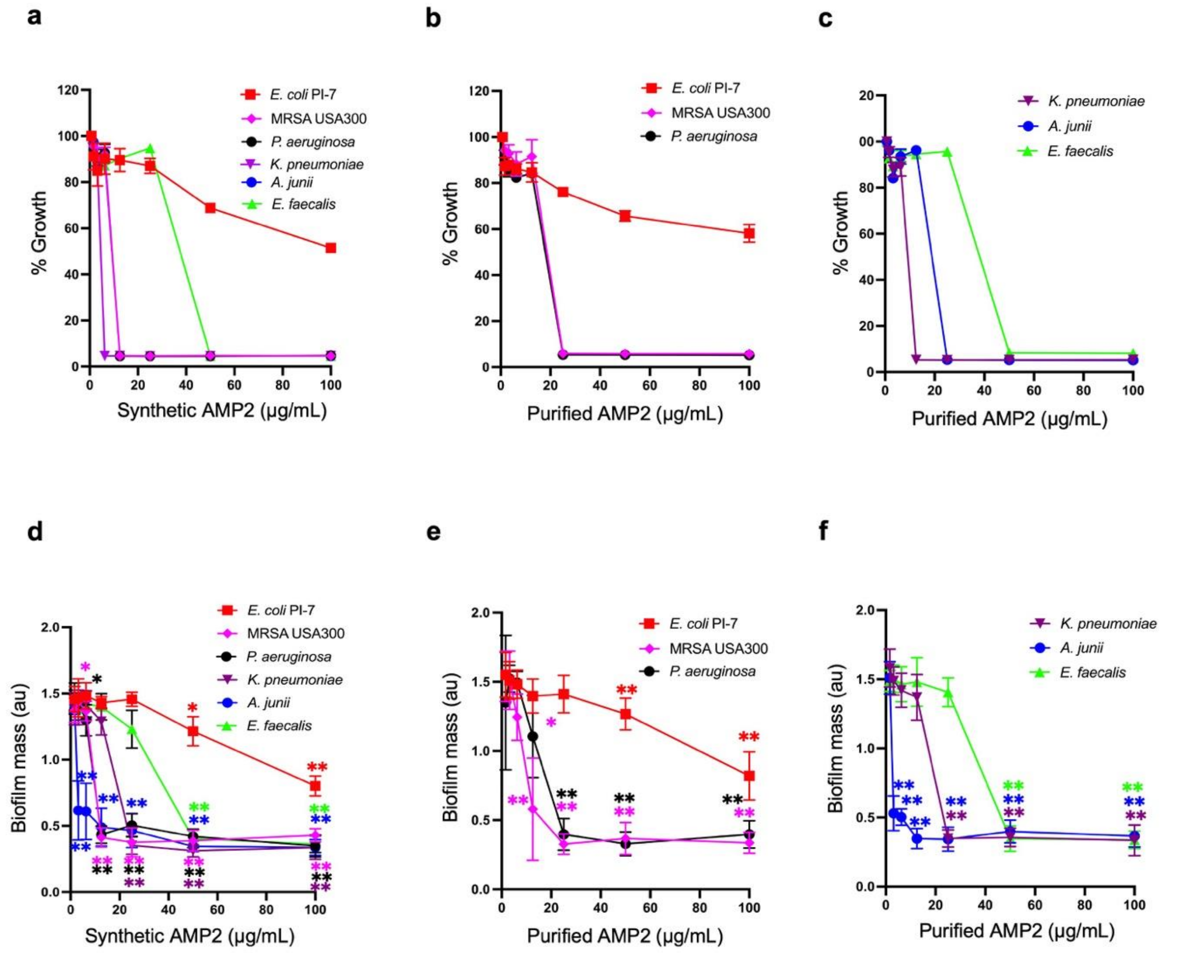

Supplementary Fig. 4. Experimental validation of antimicrobial activity of plant purified AMP2 against ESKAPE pathogens and their prevention of biofilm formation.

a-d, Plant-produced and synthetic peptides have the same efficacy in bacterial growth inhibition. For each concentration of peptide, 10^6 colony-forming units (CFU)/mL of each ESKAPE pathogens were treated with 1=100 µg/mL, 2=50 µg/mL, 3=25 µg/mL, 4=12.5 µg/mL, 5=6.25 µg/mL, 6=3.215 µg/mL, 7=1.56 µg/mL of peptides in cation-adjusted Mueller-Hinton broth (Ca-MHB) for 24 h. Percentage inhibition was up to >40% on carbapenem-resistant *E. coli* PI-7 in OD600, at a concentration of pp: 50µg/mL, sp: 50 µg/mL, >90% of inhibition of MRSA USA300 (pp: 25 µg/mL, sp: 12.5 µg/mL), *K. pneumoniae* (pp: 25 µg/mL, sp: 6.25 µg/mL), *A. junii* (pp: 25 µg/mL, pp: 12.5 µg/mL), *E. faecalis* (pp: 50 µg/mL, sp: 50 µg/mL), *P. aeruginosa* (pp: 25 µg/mL, sp: 12.5 µg/mL). pp, plant-purified peptide; sp, synthetic peptide. Data are means ± SD of two independent experiments performed in duplicates. **e-h,** Bactericidal activity of purified peptides against prevention of biofilms after 24 h of incubation in biofilm media containing various concentrations of peptides. Results are expressed as biofilm mass, measured using crystal violet staining, in arbitrary units (au). Values are medians of two independent experiments. *, significantly different (*$P < 0.05$, **$P < 0.01$, and ***$P < 0.001$) compared to control (0 µg/mL), as calculated using the two-tailed Mann-Whitney rank sum test. Source data are provided as a Source Data file.

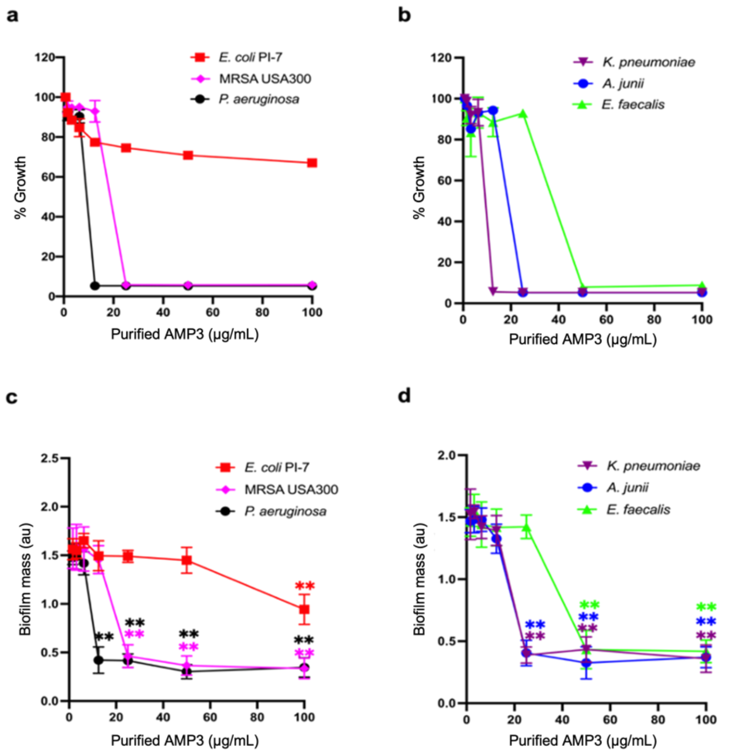

Supplementary Fig. 5. Experimental validation of antimicrobial activity of plant purified peptide AMP3 against ESKAPE pathogens and their prevention of biofilm formation.

a,b, Plant-produced peptides are effective against ESKAPE pathogens. For each concentration of peptide, 10^6 colony-forming units (CFU)/mL of each ESKAPE pathogens were treated with 100, 50, 25, 12.5, 6.25, 3.215, 1.56 µg/mL of peptides in cation-adjusted Mueller-Hinton broth for 24 h. Percentage inhibition was up to 30% reduction for carbapenem-resistant *E. coli* PI-7 in OD600, at a concentration of pp: 50 µg/mL, >90% of inhibition of MRSA USA300 (pp: 25 µg/mL), *K. pneumoniae* (pp: 25 µg/mL), *A. Junii* (pp: 25 µg/mL), *E. faecalis* (pp: 50 µg/mL), *P. aeruginosa* (pp: 12.5 µg/mL). pp, plant-purified peptide. Data are means ± SD of two independent experiments performed in duplicates. **c,d,** Bactericidal activity of purified peptides against prevention of biofilms after 24 h of incubation in biofilm medium containing various concentrations of peptides. Results are expressed as biofilm mass, measured using crystal violet staining, in arbitrary units (au). Values are medians of two independent experiments. *, significantly different (**P* < 0.05, ***P* < 0.01, and ****P* < 0.001) compared to control (0 µg/mL), as calculated using the two-tailed Mann-Whitney rank sum test. Source data are provided as a Source Data file.

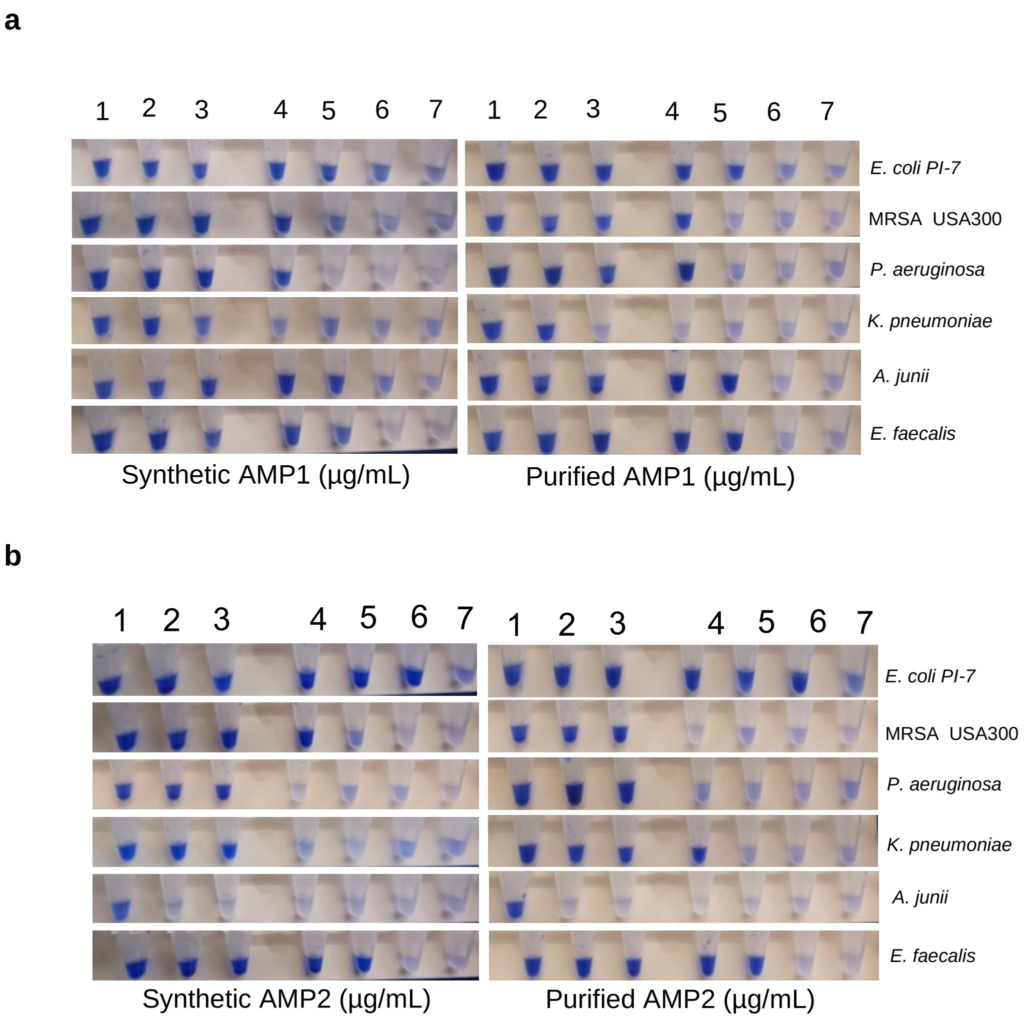

Supplementary Figure 6. Crystal violet staining assay for biofilm quantification.

a, b, 1 x 10^6 colony-forming units (CFU)/mL of each ESKAPE (*E. coli PI-7*, MRSA USA300, *P. aeruginosa*, *K. pneumoniae*, *A. junii*, *E. faecalis*) pathogen were treated with 1=100 µg/mL, 2=50 µg/mL, 3=25 µg/mL, 4=12.5 µg/mL, 5=6.25 µg/mL, 6=3.215 µg/mL, 7=1.56 µg/mL of peptides in cation-adjusted Mueller-Hinton broth for 24 h, biofilms were stained with 1% [w/v] crystal violet. The bounded crystal violet stain was dissolved in 95% [v/v] ethanol. Source data are provided as a Source Data file.

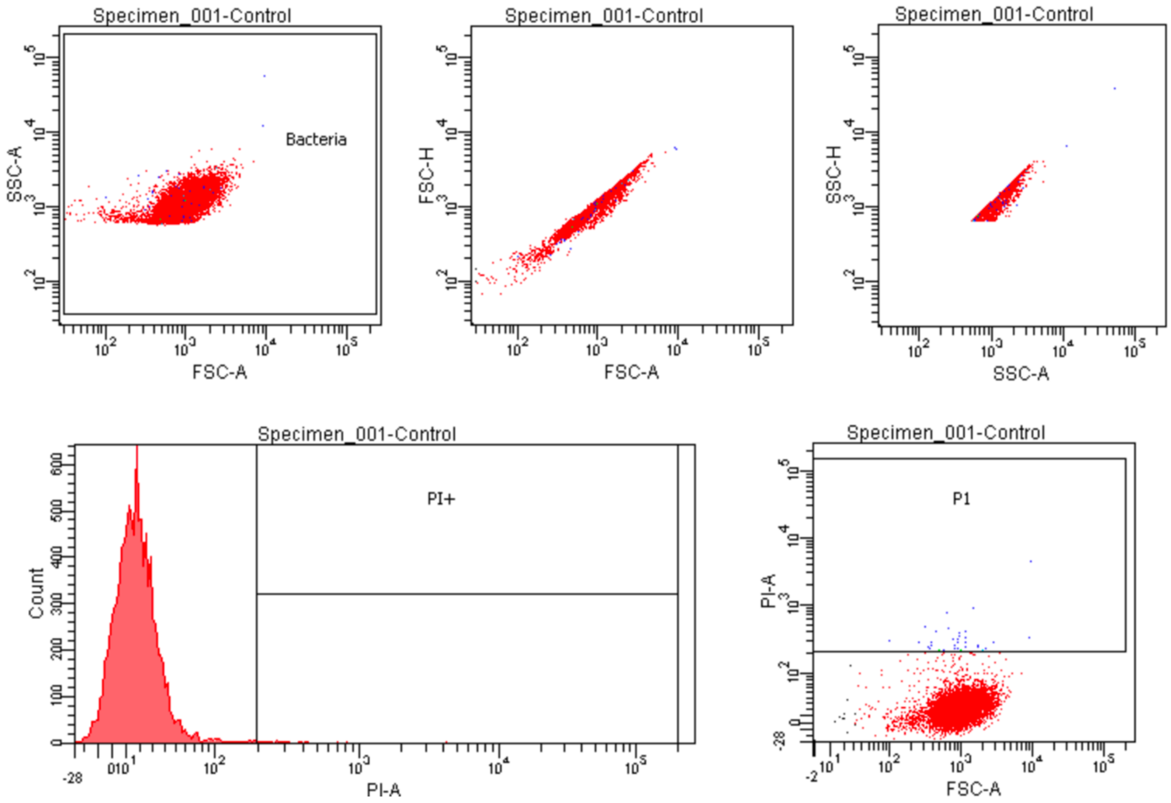

Supplementary Fig. 7. Gating strategy for flow cytometry-based analysis of PI accumulation in MRSA USA300 cells

Mid-logarithmic growth-phase culture of MRSA USA300 (OD 600 = 0.5 = 10^8 CFU cells/mL) without any treatment were interrogated with 25 μL of 1 mg/mL propidium iodide for 30 mins in dark. Cells were washed, suspended in 500 μL of 1 x PBS and analyzed on BD LSRFortessa™ Cell Analyzer. Cells were gated on forward and side scatter profiles. Positive and negative cell populations were gated based on staining the fluorescently-PI dye. At least 1000 events were analyzed. Control cells showed negligible or very low accumulation of PI stain.

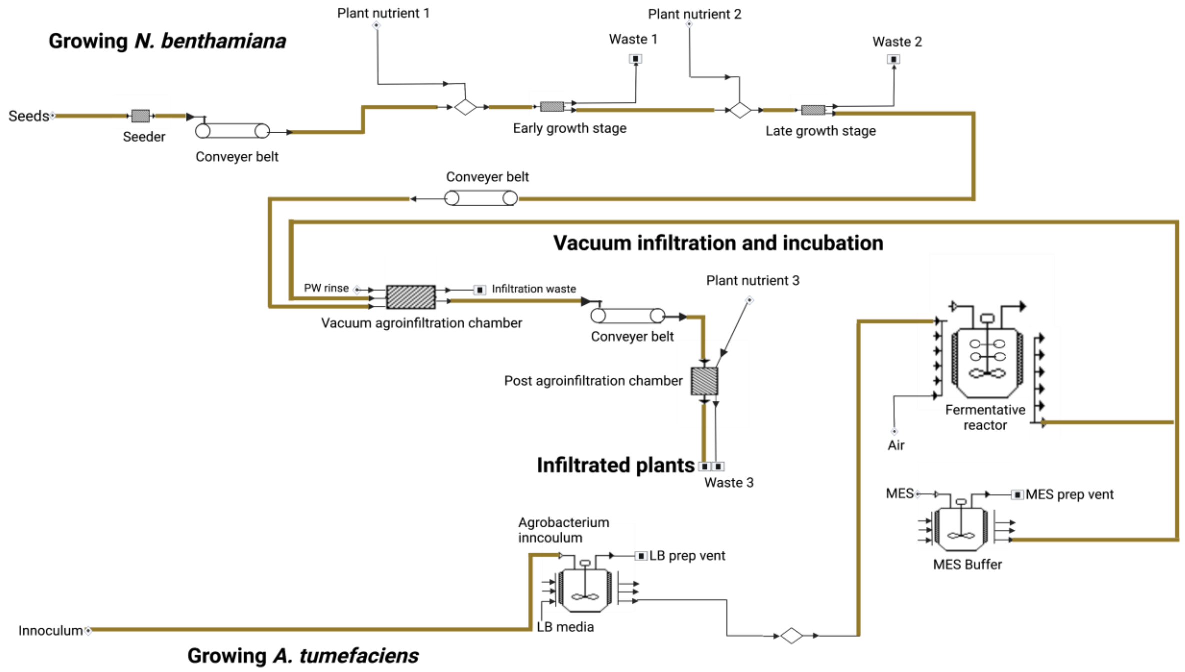

Supplementary Fig. 8. Base-case techno-economic analysis for industrial scale production of AMPs in plants.

Upstream process flowsheet for *N. benthamiana* base case scenario in the SuperPro Designer model with a production capacity at 300 Kg/year. Source data are provided as a Source Data file.

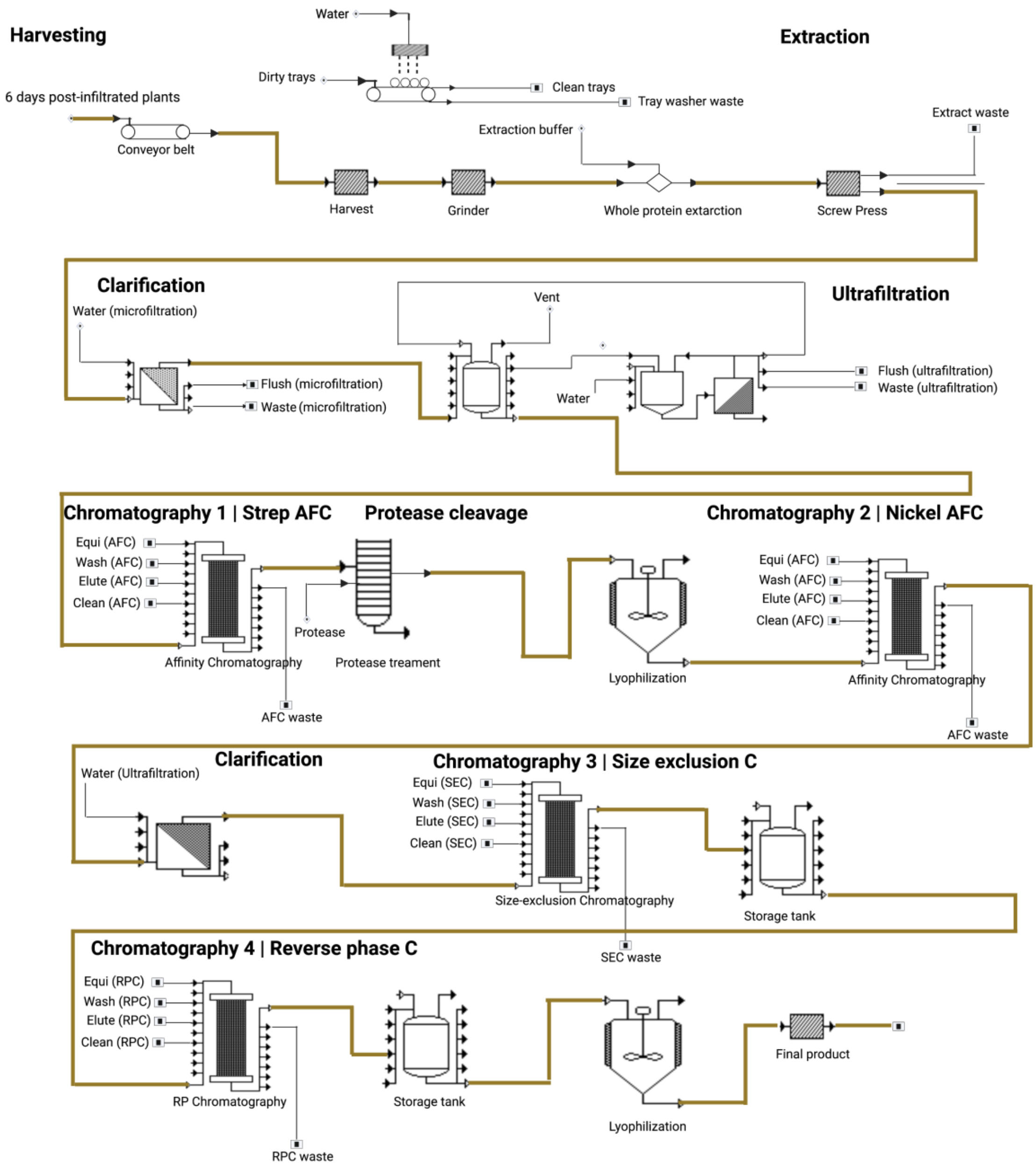

Supplementary Fig. 9. Base-case techno-economic analysis for industrial scale production of AMPs in plants.

Downstream process flowsheet for *N. benthamiana* base case scenario in the SuperPro Designer model with a production capacity at 300 Kg/year. Source data are provided as a Source Data file.

Supplementary Table 1. Antimicrobial activity of plant purified AMP1 peptide.

**Organisms**	**Synthetic AMP1 (MIC µg/mL)**	**Purified AMP1 (MIC µg/mL)**
E. coli PI-7	50	50
MRSA US300	25	25
P. aeruginosa	25	25
K. pneumoniae	6.25	6.25
A. junii	12.5	50
E. Faecalis	25	25

E. coli PI-7: Escherichia coli PI-7; MRSA USA300: Methicillin resistant Staphylococcus aureus USA300; P. aeruginosa: Pseudomonas aeruginosa; K. pneumoniae: Klebsiella pneumoniae; A. junii: Acinetobacter junii; E. faecalis: Enterobacter faecalis

Supplementary Table 2. Antimicrobial activity of plant purified AMP2 peptide.

**Organisms**	**Synthetic AMP2 (MIC µg/mL)**	**Purified AMP2 (MIC µg/mL)**
E. coli PI-7	>50	>50
MRSA US300	12.5	25
P. aeruginosa	12.5	25
K. pneumoniae	6.25	12.5
A. junii	12.5	25
E. Faecalis	50	50

E. coli PI-7: Escherichia coli PI-7; MRSA USA300: Methicillin resistant Staphylococcus aureus USA300; P. aeruginosa: Pseudomonas aueruginosa; K. pneumoniae: Klebsiella pneumoniae; A. junii: Acinetobacter junii; E. faecalis: Enterobacter faecalis

Supplementary Table 3. Unit cost of electricity, labor and utilities referred to the Saudi Arabian.

Operator labor	24.74 $\$hr^{-1}$
Electricity	0.068 $ $kWhr^{-1}$
Hot water	2 $ MT^{-1}
Cold water	0.25 $ MT^{-1}
Steam	12.2 $ MT^{-1}

Supplementary Table 4. Table showing economic capital investment, operating expenditures (with and without depreciation) and calculated the cost of goods sold (COGS) for plant-based AMP production scenario.

	**Upstream**	**Downstream**	**Total**
Total capital investment in millions (USD)	$43	$78.60	$121.60
Total annual operating cost including depreciation in millions USD (% of total)	$13.20	$18.49	$31.69
Total annual operating cost excluding depreciation in millions USD (% of total)	$9.43	$13.21	$22.64
Cost of goods sold including depreciation ($ g/AMP)	$43	$62.00	$105.00
Cost of goods sold excluding depreciation ($ g/AMP)	$32	$42.00	$74

Supplementary Table 5. Prices of reagents used in the production of peptides adapted from Sigma-Aldrich and GE Healthcare, Europe, GmbH.

**Reagents**	**Cost (USD)**
Triton X 100 (5 mL)	44.94
NaCl (25 g)	33.49
EDTA (500 mg)	42.16
D-desthiobiotin (1 mg)	268.14
Strep-Tactin resin (10 mL)	860
Imidazole (500 mg)	211.64
Glycerol (500 mL)	167.64
Nickel NTA affinity resin (100 mL)	868
Sephadex G-10 (500 mg)	1383
Acetonitrile (1L)	201.37
Bulk C8 resin (50 mg)	390.02
HCl (2.5 L)	252.24
NaOH (1 kg)	55.54
Tryptone (1 kg)	618.95
Yeast extract (500 mg)	175.94
Rifampicin (1 mg)	170.64
Kanamycin (25 mg)	671.94
Gentamycin (250 mg)	129.3
Urea (1 kg)	102.91
Protease plasmid addgene	65
Murashige and Skoog BM (50 L)	295.70

Supplementary Table 6. Table showing economic capital investment, operating expenditures (with and without depreciation) and calculated the cost of goods sold (COGS) for plant-based AMP production scenario.

**Platform**	**COGS**	**Reference**
Solid phase synthesis	$95.29/mg	n/a
E. coli produced peptides (5 L) fermenter	$44.5-$268.16/mg	[1]
E. coli produced commercial enzyme (kg) 88 tonnes	$316/kg	[2]
Mammalian cells produced protein (250-300 kg/year)	$1450/g	[3]
Plants produced peptides (300 kg/year)	$74/g (our data)	n/a

Supplementary references

1 Gaglione, R. *et al.* Cost-effective production of recombinant peptides in *Escherichia coli*. *N Biotechnol* **51**, 39-48 (2019).

2 Ferreira, R. D. G., Azzoni, A. R. & Freitas, S. Techno-economic analysis of the industrial production of a low-cost enzyme using *E. coli*: the case of recombinant beta-glucosidase. *Biotechnol Biofuels* **11**, 81 (2018).

3 Nandi, S. *et al.* Techno-economic analysis of a transient plant-based platform for monoclonal antibody production. *MAbs* **8**, 1456-1466, (2016).
