# Supplementary material for: Efficient in planta production of amidated antimicrobial peptides that are active against drug-resistant ESKAPE pathogens
Source: Nat Commun. 2023 Mar 16;14:1464. doi: 10.1038/s41467-023-37003-z (PMC10020429; doi:10.1038/s41467-023-37003-z)
Supplement: Supplementary file 2 — Description of Additional Supplementary Files [file 41467_2023_37003_MOESM2_ESM.pdf]

Description of Additional Supplementary Files

File Name: Supplementary Data 1
Description: gBlocks sequences in blue: Strep-tag II, Red: HA epitope, Purple: flexible linker, Orange: mutated SUMO domain, dark blue: respective AMP sequences.

File Name: Supplementary Data 2
Description: Primers used in the study.
