# Supplementary material for: Efficient in planta production of amidated antimicrobial peptides that are active against drug-resistant ESKAPE pathogens
Source: Nat Commun. 2023 Mar 16;14:1464. doi: 10.1038/s41467-023-37003-z (PMC10020429; doi:10.1038/s41467-023-37003-z)
Supplement: Supplementary file 5 — Reporting Summary [file 41467_2023_37003_MOESM5_ESM.pdf]

nature portfolio

Corresponding author(s): Magdy Mahfouz

Last updated by author(s): Feb 13, 2023

Reporting Summary

Nature Portfolio wishes to improve the reproducibility of the work that we publish. This form provides structure for consistency and transparency in reporting. For further information on Nature Portfolio policies, see our Editorial Policies and the Editorial Policy Checklist.

Statistics

For all statistical analyses, confirm that the following items are present in the figure legend, table legend, main text, or Methods section.

n/a	Confirmed	
☐	☒	The exact sample size (*n*) for each experimental group/condition, given as a discrete number and unit of measurement
☐	☒	A statement on whether measurements were taken from distinct samples or whether the same sample was measured repeatedly
☐	☒	The statistical test(s) used AND whether they are one- or two-sided<br>*Only common tests should be described solely by name; describe more complex techniques in the Methods section.*
☒	☐	A description of all covariates tested
☒	☐	A description of any assumptions or corrections, such as tests of normality and adjustment for multiple comparisons
☐	☒	A full description of the statistical parameters including central tendency (e.g. means) or other basic estimates (e.g. regression coefficient) AND variation (e.g. standard deviation) or associated estimates of uncertainty (e.g. confidence intervals)
☐	☒	For null hypothesis testing, the test statistic (e.g. *F*, *t*, *r*) with confidence intervals, effect sizes, degrees of freedom and *P* value noted<br>*Give P values as exact values whenever suitable.*
☒	☐	For Bayesian analysis, information on the choice of priors and Markov chain Monte Carlo settings
☒	☐	For hierarchical and complex designs, identification of the appropriate level for tests and full reporting of outcomes
☒	☐	Estimates of effect sizes (e.g. Cohen's *d*, Pearson's *r*), indicating how they were calculated

Our web collection on statistics for biologists contains articles on many of the points above.

Software and code

Policy information about availability of computer code

Data collection	UNICORN 6.3 (Build 6.3.2.89) GE Life Science;<br>SuperPro Designer Version 13 (Intelligen, Inc);<br>Tecan i-control 2 (2.0.10.0) Austria, GmbH;<br>Agilent Open LAB CDS Chemstation C.01.07 SR2 (55), Agilent Technologies;<br>Compass for otofSeries 1.7 Version 3.4 (Bruker Daltonics GmbH);<br>ImageQuant LAS 4000, Version 1.0, GE Healthcare;<br>Image Lab Version 6.0.1, Bio-Rad Laboratories;<br>SmartSEM Version 6.09, Serial Number Merlin-61-95 Carl Zeiss Microscopy;<br>Leica Application Suite X 3.5.5.19976;<br>BD FACSDiva Software, Version 6.2 (build 2010 03 29 11 08), Firmware Version 1.12 (BD LSR II) (BD Biosciences).
Data analysis	Agilent Open LAB CDS Chemstation C.01.07 SR2 (55), Agilent Technologies;<br>Bruker Compass DataAnalysis 4.2 SR2, Bruker Daltonics GmbH;<br>Affinity designer Version 1.9.3 (Serif labs);<br>GraphPad Prism Version 9.4.1 (458);<br>FlowJo (Version 10.6.2) (BD Biosciences);<br>all analysis was carried out using Microsoft Excel Version 16.54 (21101001).

For manuscripts utilizing custom algorithms or software that are central to the research but not yet described in published literature, software must be made available to editors and reviewers. We strongly encourage code deposition in a community repository (e.g. GitHub). See the Nature Portfolio guidelines for submitting code & software for further information.

Data

Policy information about availability of data

All manuscripts must include a data availability statement. This statement should provide the following information, where applicable:

- Accession codes, unique identifiers, or web links for publicly available datasets
- A description of any restrictions on data availability
- For clinical datasets or third party data, please ensure that the statement adheres to our policy

Data availability statement has been added to the revised mansucript. All data supporting the findings of this study are available within the paper (and supplementary information files). Source data are provided with this paper. Figure 1b was created using Affinity designer Version 1.9.3 (https://affinity.serif.com/en-us/) and Extended Data Fig. 6 using SuperPro Designer® 13.0 software (https://www.intelligen.com). Public database used for rat PAM enzyme sequence include UniProt (https://www.uniprot.org/uniprotkb/A0A8I5ZMR1/entry).

Human research participants

Policy information about studies involving human research participants and Sex and Gender in Research.

Reporting on sex and gender	N/A
Population characteristics	N/A
Recruitment	N/A
Ethics oversight	N/A

Note that full information on the approval of the study protocol must also be provided in the manuscript.

Field-specific reporting

Please select the one below that is the best fit for your research. If you are not sure, read the appropriate sections before making your selection.

[x] Life sciences [ ] Behavioural & social sciences [ ] Ecological, evolutionary & environmental sciences

For a reference copy of the document with all sections, see nature.com/documents/nr-reporting-summary-flat.pdf

Life sciences study design

All studies must disclose on these points even when the disclosure is negative.

Sample size	No statistical methods were used to predetermine sample size. All exerimental data were conducted on three or more indepedent experiments to esnure each data point was reproducible.
Data exclusions	No data were excluded.
Replication	Number of experimental replicates are displayed in the figure legends. All attempts at replication were successful.
Randomization	All the experimental data quantification and analysis was done using standard software packages, and are unbiased. Therefore, no randomization was needed to perform.
Blinding	All the experimental data quantification and analysis was done using standard software packages, and are unbiased. Therefore, no blinding was needed to perform.

Reporting for specific materials, systems and methods

We require information from authors about some types of materials, experimental systems and methods used in many studies. Here, indicate whether each material, system or method listed is relevant to your study. If you are not sure if a list item applies to your research, read the appropriate section before selecting a response.

Materials & experimental systems

n/a	Involved in the study
[ ]	[x] Antibodies
[ ]	[x] Eukaryotic cell lines
[x]	[ ] Palaeontology and archaeology
[x]	[ ] Animals and other organisms
[x]	[ ] Clinical data
[x]	[ ] Dual use research of concern

Methods

n/a	Involved in the study
[x]	[ ] ChIP-seq
[ ]	[x] Flow cytometry
[x]	[ ] MRI-based neuroimaging

Antibodies

Antibodies used

1. Anti-HA high affinity, Sigma-Aldrich, clone name: 3F10, catalog no: 11867431001, diluted to 1:1000 for WB application (produced in rat).

2. Goat Anti-Rat IgG antibody HRP conjugate, Sigma-Aldrich, SKU number: AP136P diluted to 1:5,000 for WB application (produced in Goat).

3. Anti-GFP antibody, Abcam, lot number: ab6556, polyclonal, diluted to 1:1500 for WB application (produced in rabbit).

4. Goat Anti-Rabbit IgG H&L (HRP), lot number: ab205718 diluted to 1:2000 for WB application (produced in goat).

Validation

1."Anti-HA high affinity is a high-affinity monoclonal antibody that can be used to detect HA-tagged proteins." (https://www.sigmaaldrich.com/SA/en/product/roche/roahaha).

2. "Goat Anti-Rat IgG is highly specific for rat IgG, heavy and light chain." (https://www.sigmaaldrich.com/SA/en/product/mm/ap136p).

3. "Anti-GFP detects the GFP fraction from cell extracts expressing recombinant GFP fusion proteins". (https://www.abcam.com/gfp-antibody-ab6556.html).

4. "Goat Anti-Rabbit IgG is used for conjugation reacts with rabbit immunoglobulins of all classes." (https://www.abcam.com/goat-rabbit-igg-hl-hrp-ab205718.html).

Eukaryotic cell lines

Policy information about cell lines and Sex and Gender in Research

Cell line source(s)

Human embryonic kidney cell (HEK 293) was purchased from ThermoFisher Scientific, (cat. No. 51-0035, lot number 1848145).

Authentication

We didn't performed any DNA profile to confirm the authenticity of the cell line.

Mycoplasma contamination

Mycoplasma was checked every 2-3 months and was found to be negative in all cell lines used.

Commonly misidentified lines (See ICLAC register)

The cell line is not listed in the database of commonly misidentified cell lines.

Flow Cytometry

Plots

Confirm that:

- [x] The axis labels state the marker and fluorochrome used (e.g. CD4-FITC).
- [x] The axis scales are clearly visible. Include numbers along axes only for bottom left plot of group (a 'group' is an analysis of identical markers).
- [x] All plots are contour plots with outliers or pseudocolor plots.
- [x] A numerical value for number of cells or percentage (with statistics) is provided.

Methodology

Sample preparation

Pick 3-4 colonies of MRSA USA300 bacteria and grow overnight at 37 degree in Tryptic soy broth containing 10 µg/ml chloramphenicol.

Next day, dilute the cells to 1:50 and grow till mid-logarithmic phase (OD 600 = 0.5 = 10^8 CFU cells/ml) in Tryptic soy broth containing 10 µg/ml chloramphenicol. It usually take 2 hrs for bacteria to attain the mid-logarithmic phase.

After 2 hrs, take 1.5 ml of the cells and resuspend in 1.5 ml of Mueller-Hinton broth correlating to 1 x 10^8 CFU cells/ml.

Treat cells with vancomycin (for 2.5 hr), 70% ethanol as positive control (for 1 hr), non-treated bacteria (as control), purified AMP1 (for 1 hr).

After the mentioned time-point, interrogate with 25 ul of 1 mg/ml Propidium iodide and stain in dark for 30 mins.

Wash with 1 x PBS and suspend the pellet in 500 ul of 1 x PBS buffer.

Instrument	BD LSRFortessaTM Cell Analyzer (BD Biosciences, San Jose, CA, USA)
Software	BD FACSDiva Software, Version 6.2 (build 2010 03 29 11 08) was used to perform flow cytometry and FlowJo (Version 10.6.2) was used for flow cytometry data analysis.
Cell population abundance	We have used homogenous population of drug-resistant MRSA USA300 bacterial cells and sorting or enrichment was not required. Atleast 1000 events were analyzed.
Gating strategy	MRSA USA300 bacterial cells were gated on forward and side scatter profiles. Positive and negative cell populations were gated based on staining the fluorescently-PI dye.

☒ Tick this box to confirm that a figure exemplifying the gating strategy is provided in the Supplementary Information.
